# Relationship Value, Emotion, and Uncertainty Shape Conflict−Resolution Communication in a Wild Primate

**DOI:** 10.1111/nyas.70352

**Published:** 2026-07-30

**Authors:** Luca Pedruzzi, Alice Galotti, Martina Francesconi, Alberto Quartesan, Sheleme Abiyou Gamessa, Hailu Tilahun Argaw, Arianna Meletiadis, Pier Luigi Acutis, Maria Vittoria Riina, Valentina Serra, Giulio Petroni, Bezawork Afework Bogale, Alban Lemasson, Elisabetta Palagi

**Affiliations:** ^1^ Centre D'etude En Ethologie et Cognition (CEEC) ‐ U.M.R 6552 Université De Rennes, Université de Normandie, CNRS Rennes France; ^2^ Unit of Ethology, Department of Biology University of Pisa Pisa Italy; ^3^ Department of Zoological Sciences Addis Ababa University Addis Ababa Ethiopia; ^4^ Istituto Zooprofilattico Sperimentale Del Piemonte, Liguria e Valle D'aosta Torino Italy

## Abstract

Studying how nonhuman primates tailor signals and behavioral patterns to resolve ingroup conflicts provides insight into the evolution of communicative and behavioral complexity in conflict management. Here, we explore how, in wild geladas, primates with a uniquely rich vocal−visual repertoire, aggressors and victims take into account relationship value, perceived risk and uncertainty, emotional dynamics, and audience and external factors when producing nonaggressive signals after conflicts. We found that reconciliation in the study groups occurred rapidly, was mainly initiated by aggressors, and was mediated by post‐conflict sequences composed of vocalizations, sometimes supplemented with visual signals and tactile patterns. The complexity of post‐conflict sequences—indexed by their length, sensory channels, and signal types—was amplified in aggressor−victim dyads with stronger social bonds, following more intense victim distress signals, with dominant aggressors, and between genetically unrelated individuals. These patterns reflect sensitivity to uncertainty, relationship quality, emotional states, and to the external audience. Such behavioral flexibility helps individuals navigate the challenges of complex social life, where multiple relationships must be continuously tracked, thereby supporting social competence and group cohesion. Post‐conflict signaling lies at the intersection of social and cognitive sophistication, revealing evolutionary links to human complex communication.

## Graphical abstract

Geladas combine vocal−visual signals and tactile patterns to resolve conflicts. Reconciliation is rapid, mainly aggressor‐initiated, and shaped by relationship value, risk, emotion, and audience context. Post‐conflict sequences increase in complexity with stronger bonds, greater distress, dominance asymmetries, and genetic unrelatedness. These patterns highlight how social complexity drives flexible conflict−resolution strategies and its evolutionary links to human communication.

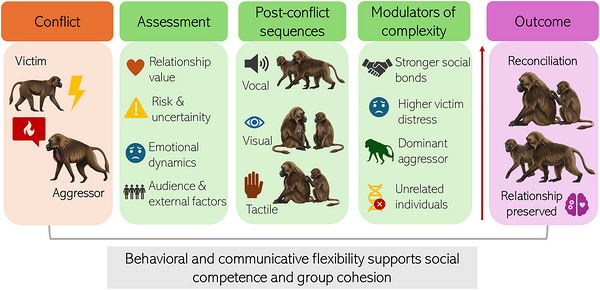

## 1 Introduction

In group‐living animals, communication relies on a range of cues and signals (e.g., vocalizations, facial expressions, body postures) produced and exchanged with varying degrees of flexibility and strategic use [1, 2, 3]. Despite the ongoing debate over whether nonhuman communication is primarily intentional or spontaneous [4−6], growing evidence indicates at least some control over signal production [7]. Although nonhuman animals generally use relatively constrained repertoires [8], they may enhance variability through other means—such as modulating signal intensity and diversity, or combining modalities—to convey more graded information [9, 10, 11, 12, 13, 14]. Complex communication—reflected in both larger and more flexibly deployed signal repertoires [15, 16]—often covaries with the socioecological complexity of a species or population (hereafter, social complexity).

At the individual level, social complexity can be conceived as the number of differentiated social relationships an individual forms and maintains, with differentiation increasing when partners are differently treated according to relative rank, kinship, age, or interaction history [17, 18]. Increasing relational variability is often reflected in the flexibility and richness of signaling behavior: interacting with partners that differ in dominance rank, kinship, and shared history generates social uncertainty, making interaction outcomes less predictable and requiring individuals to adjust their signaling strategies to reduce this uncertainty [9, 19]. For instance, animals can flexibly tailor the use of signals according to the perceived interaction social uncertainty or risk deriving from partner identity or audience composition [9, 20, 21]. To enhance flexibility, communicative sequences can be modified in physical form by varying the types, intensity, and modality of signals during the interaction. Changes in physical form may also lead to alterations in the social functions of both individual and combined signaling behaviors [9]. These changes can be sensitive to the relationship history with the interacting partner, their dominance ranking, but also to other contextual features such as the possible influence of nearby uninvolved individuals [22]. The capacity to recognize and respond to behaviors reflective of underlying emotional states [23], can also shape how individuals flexibly deploy signals. Sensitivity to receivers’ affective states enables real‐time adjustment of signaling, including persistence, intensification, or modality switching to convey positive intent or reassurance [7, 24, 25, 26].

Going from the individual to the group level, richer and more flexible signal repertoires are associated with larger group sizes, increased demands for social bonding, and more tolerant systems characterized by more uncertain interactions [10, 11, 20, 27, 28]. Communicative complexity can thus vary across groups with different sociobiological systems [11, 15, 28, 29, 30, 31], or within a group across demographic categories—for instance across developmental stages [32, 33] or between males and females [34, 35]—when these individuals experience differently complex social environments.

**FIGURE 1:**
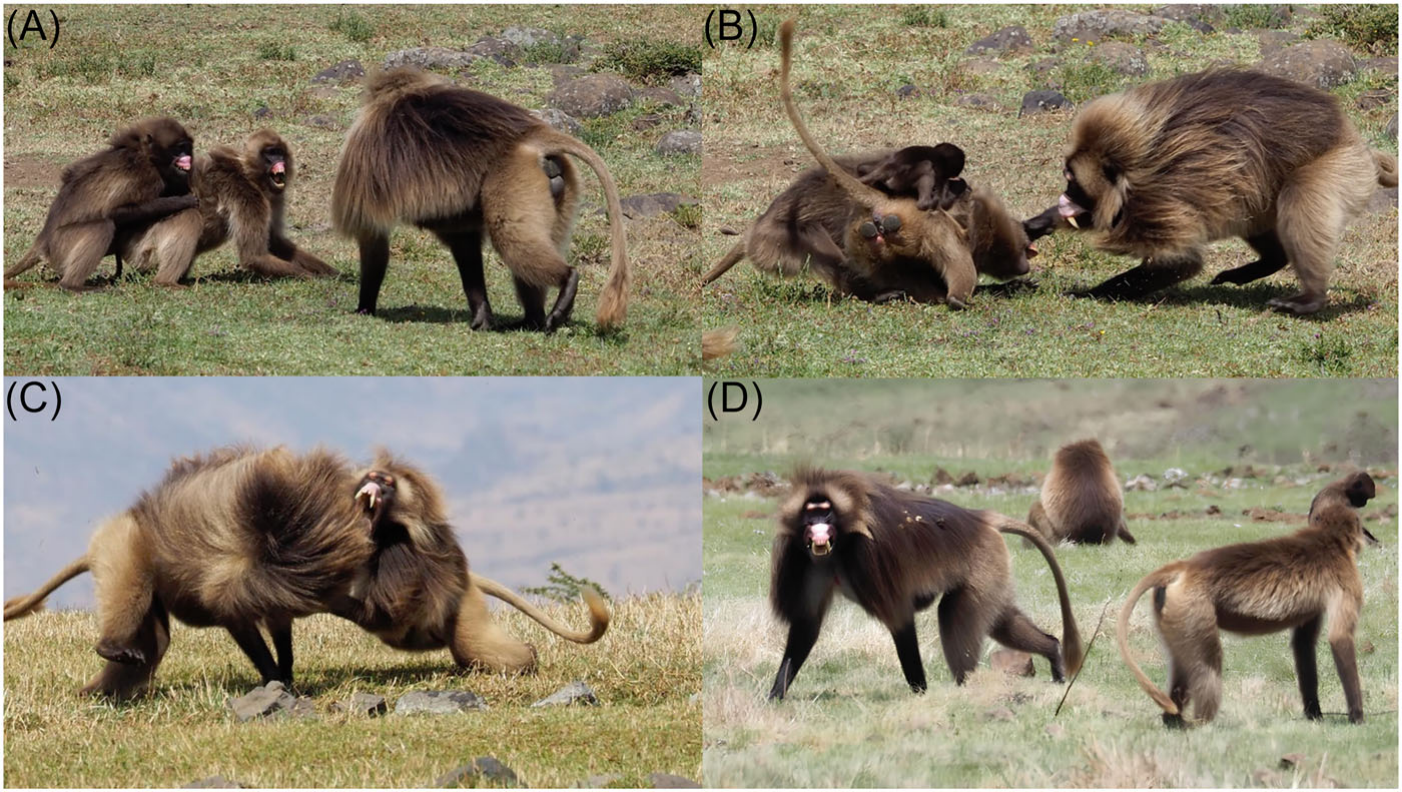
Pictures of geladas in Debre Libanos (Ethiopia), with a male attacking two females (A−B), male−male aggression (C), and a male producing a bared‐teeth with lip‐flip movement and eyebrow raiser to a female (D). Pictures taken by Alice Galotti and Martina Francesconi.

Studying how primates adjust communication according to past experience, contextual factors, and emotional feedbacks from the partner reveals the cognitive and affective bases of communication [9, 22, 36]. A fruitful arena for studying these aspects is aggression (Figure 1), a context of heightened emotional arousal that threatens relationship and group stability [37]. Individuals often engage in intensified communicative efforts to mitigate conflict with potentially detrimental consequences [9, 20, 33, 38]. Several behavioral strategies have evolved to manage aggression [37, 39]. Among these, reconciliation represents the first exchange of nonaggressive patterns between former aggressors and victims occurring earlier after conflicts compared to control conditions [37]. Reconciling can reduce physiological stress [40] and restore relationships and social benefits [38, 41]. Although reconciliation was traditionally considered to occur through physical affiliation [42], subsequent research has shown that nonaggressive vocal [43, 44, 45, 46, 47] and visual signals [48] can also be exchanged in the aftermath of conflicts, highlighting the importance of a multimodal approach [49]. These signals may not necessarily function to repair a damaged relationship; they could communicate nonaggressive intentions and reduce uncertainty about whether aggression will resume, thereby facilitating subsequent peaceful interactions. Recent work has encouraged the closer examination of acoustic signaling in animal post‐conflict interactions [45], noting that vocalizations—effective over distance and involving lower risk—may function as grooming at a distance [50, 51]. Vocalizations can signal that a conflict has ended and may facilitate peaceful post‐conflict interactions, such as grooming, thereby helping to restore pre‐conflict social conditions. The signals and patterns exchanged after conflicts can be influenced by the dyadic relationship (e.g., social bonds, kinship, dominance) and by contextual factors such as perceived risk and uncertainty [38, 45, 52, 53, 54, 55]. While the presence of reconciliation has been extensively studied in primates, the communicative flexibility deployed in victim−aggressor post‐conflict signaling remains relatively understudied.

Among nonhuman primates, geladas (*Theropithecus gelada*) are characterized by rich vocal and visual repertoires, embedded within highly complex social systems [29, 56]. Geladas are monkeys endemic to Ethiopian Afro‐alpine grasslands [57] and living in multilevel societies—an intricate form of social organization characterized by two or more nested levels of organization [56]. The core units are typically composed of one reproductive leader male, 1−12 philopatric and related females [58], their offspring, and possibly subordinate males [56]. Despite their graminivorous diet, geladas display linear dominance hierarchies and show competition over some food resources [58, 59]. Although levels of relatedness among females within units are generally high, females still bond preferentially with the closest kin [60]. Intragroup dynamics in geladas are marked by strong and enduring male−female bonds, often lasting several years [56, 61]. Aggression is part of intragroup dynamics (Figure 1): females can compete for resources, but preserving female−female bonds is essential to obtain agonistic support and increase offspring survival [62, 63]; female−female bonds can even grant cohesion in the extreme case of leader male loss [56, 62]. While male aggression toward females is frequent to avoid extra‐group mating in a multigroup environment [64, 65], males maintain cohesion with adult females through affiliation and not coercion [29], making male motivation to solve conflicts highly relevant. In this direction, the need to maintain these cross‐sex bonds has likely shaped male distinctive communicative repertoire—one marked by unique signals absent in closely related primates [29, 35, 66, 67]. Indeed, derived signals include derived affiliative vocalizations (e.g., moans, wobbles) thought to have evolved as a means of sustaining cross‐sex bonds and promoting unit cohesion without relying on coercive strategies [35]. While captive geladas seem to use vocalizations, facial expressions, and physical contact (grooming) to reconcile their conflicts [68, 69], recent work failed to find evidence for this phenomenon in the wild [70], raising questions on intraspecific variability of conflict−management strategies. Although some studies show that geladas deploy different signals in emotionally tense interactions [29, 68, 71] and recognize vocal affiliation toward distressed victims [72], research explicitly investigating gelada aggressor−victim interactions and signaling remains limited.

Here, we examine the interactional variables shaping the exchange of nonaggressive patterns and signals between the victim and aggressor after conflicts (hereafter, post‐conflict sequences). Post‐conflict sequences comprise vocalizations, visual signals (e.g., facial expressions, gestures), and patterns of physical contact (e.g., body contact, grooming) traditionally examined in post‐conflict interactions (see Methods). The complexity of sequences is indexed by their length (duration in seconds), their diversity, captured by the number of distinct signal types included within a sequence (e.g., different vocalizations, facial expressions, gestures, or tactile behaviors), and multimodality, the extent to which signals from different sensory modalities—vocal, visual, and tactile—are combined within a sequence. For example, a low‐complexity sequence may consist of a single vocalization (e.g., a single short series of grunts to the partner), whereas a high‐complexity sequence may involve multiple signals across modalities (e.g., two different types of vocalizations, a facial expression, grooming performed to the partner), reflecting both greater length, higher diversity, and multimodal integration. In parallel, we also test whether nonphysical signals can lead to subsequent physical affiliation [44].

We propose that the presence and complexity of post‐conflict sequences can be understood through four partially interdependent pillars capturing major sociocognitive challenges faced by geladas. Importantly, these pillars represent conceptual dimensions rather than mutually exclusive predictors: some variables may contribute to multiple dimensions simultaneously (e.g., dominance asymmetries may reflect both interactional risk and emotional consequences).

Relationship value reflects the adaptive importance of socially valuable relationships. In group‐living species, individuals are expected to invest more in repairing conflicts when interactions involve partners that provide significant social benefits (relationship value hypothesis, RVH). From this perspective, the presence and complexity of post‐conflict sequences should be higher in dyads with stronger affiliative bonds (Prediction RV1—Social bond strength). In addition, given the central role of male−female bonds in gelada social units—where females gain protection, and males can maintain reproductive access—we predicted that cross‐sex dyads would show particularly high reconciliation rates and communicative investment (Prediction RV2—Sex combination).

Risk and interactional uncertainty refers to situations in which the outcome of an interaction is difficult to predict or may entail high social costs. Under such conditions, individuals may deploy more elaborate communication to reduce uncertainty (risk−uncertainty hypothesis, RUH). Here, although we group risk and uncertainty under a single overarching hypothesis, we note that the first two predictions (RU1, RU2) primarily address uncertainty, whereas the third (RU3) is more directly related to risk. Interactions between nonkin or with similarly ranked partners involve higher uncertainty and lower tolerance, leading individuals to invest more effort in restoring predictability (Prediction RU1—Kinship; Prediction RU2—Rank proximity). Importantly, these two predictors are not independent in species with matrilineal dominance hierarchies, such as geladas, where kinship and rank proximity are inherently correlated. As a consequence, any positive association between reconciliation and kinship may also covary with rank distance, and the two effects should, therefore, be interpreted as partially confounded at the structural level. Finally, the intensity of the aggression (i.e., its duration, the use of intense physical contacts including bites) can influence communicative responses: severe aggression may threaten relationship stability but also increase the immediate risk of retaliation. Accordingly, intense aggression is predicted to reduce the overall probability of reconciliation but should involve more complex signaling strategies to restore the relational equilibrium (Prediction RU3—Aggression intensity).

Emotional dynamics influence both the production and perception of signals (emotional dynamics hypothesis, EMH). In conflicts, cues of victim distress offer a proximate index of emotional load [2, 73, 74, 75]. We used scream duration as a proxy of the intensity of victim distress expressed during the conflict (rather than as a direct physiological measure of internal arousal), and not as an objective measure of interaction intensity. In primates, screams are reliably produced in aversive contexts and convey graded information about the callers’ internal state, either through their acoustic parameters or their duration [76, 77]. Greater expression of distress may increase the likelihood and complexity of conciliatory communication, either because aggressors respond to the victim's emotional state or because victims seek reassurance after experiencing distress (Prediction EM1—Victim distress). Furthermore, when aggressors hold a strong dominance advantage over victims—potentially increasing fear or social vulnerability—we expected former opponents to deploy more elaborate communicative sequences to mitigate the consequences of the conflict (Prediction EM2—Dominance asymmetry).

Finally, audience and external effects (AEH hypothesis) refer to how bystanders and environmental factors can affect signaling strategies. In multilevel societies, the presence of neighboring group units may increase the potential costs of social instability or aggression spread. We thus predicted that an increasing number of neighboring units and adults in the group unit would increase the likelihood of reconciliation (Prediction AE1—Social audience size). Conversely, we expected third‐party agonistic support during aggression to reduce the need for reconciliation (Prediction AE2—Third‐party support). Finally, anthropogenic disturbance on wild groups—the presence of humans or livestock—might interfere with natural behavioral strategies and thus potentially reduce reconciliation (Prediction AE3—Anthropogenic disturbance). Table 1 summarizes our theoretical framework and how different predictions have been tested.

**TABLE 1:** Table summarizing the main hypotheses (mapping onto the four proposed pillars affecting signaling flexibility), predictions of the current study, as well as the models testing them.

Pillar/hypothesis	Prediction	Response variables (testing model)	Outcome
**Relationship value hypothesis (RVH)**. Individuals should invest more in repairing conflicts when interactions involve socially valuable partners.	**RV1—Social bond strength**. Dyads with stronger affiliative bonds reconcile more often and produce more elaborate post‐conflict sequences.	**Model 2** (probability of reconciliation) **Models 3** (sequence length), **4** (multimodality), **5** (complexity index)	**Supported** (by **Models 4 and 5**): social bond affects the multimodality and overall complexity of the post‐conflict sequence
	**RV2—Sex combination**. As cross‐sex bonds are central to gelada social units, male−female dyads are predicted to show higher reconciliation rates and greater communicative investment.	**Models 1** and **2** (latency and probability of reconciliation) **Models 3** (sequence length), **4** (multimodality), **5** (complexity index)	**Partial support** (by **Models 1** and **2**): aggression with male aggressors is more often reconciled; no effect for the sex of the second contestant
**Risk−uncertainty hypothesis (RUH)**. When interactions involve higher uncertainty or potential social costs, individuals are expected to deploy greater communicative efforts.	**RU1—Kinship**. Conflicts between unrelated individuals involve greater social uncertainty and thus lead to higher reconciliation likelihood and more elaborate post‐conflict signals.	**Model 2b** (probability of reconciliation) **Models 3b** (sequence length), **4b** (multimodality), **5b** (complexity index)	**Supported** (by **Models 3b** and **5b**): unrelated dyads exchange longer and, overall, more complex post‐conflict sequences
	**RU2—Rank proximity**. When aggressor and victim are close in rank, interaction outcomes are less predictable, sequences are expected to be more elaborate.	**Model 2** (probability of reconciliation) **Models 3** (sequence length), **4** (multimodality), **5** (complexity index)	**Not supported**
	**RU3—Aggression intensity**. More intense or prolonged aggression may reduce the overall likelihood of reconciliation, but when reconciliation occurs, it should involve more elaborate post‐conflict sequences.	**Model 2** (probability of reconciliation) **Models 3** (sequence length), **4** (multimodality), **5** (complexity index)	**Not supported**
**Emotional dynamics hypothesis (EMH)**. Emotional states generated during conflicts influence the production and reception of communicative signals.	**EM1—Victim distress**. Using victim scream duration as a proxy, higher distress is predicted to increase both the likelihood and communicative complexity of post‐conflict sequences.	**Model 2** (probability of reconciliation) **Models 3** (sequence length), **4** (multimodality), **5** (complexity index)	**Supported** (by **Model 3**): longer victim screams lead to longer post‐conflict sequences exchanged
	**EM2—Dominance asymmetry**. When aggressors hold a strong dominance advantage over victims, the latter may experience higher distress or social vulnerability; therefore, more elaborate post‐conflict communication is expected to mitigate the consequences.	**Model 2** (probability of reconciliation) **Models 3** (sequence length), **4** (multimodality), **5** (complexity index)	**Supported** (by **Models 4** and **5**): the more dominant the aggressor over the victim, the more often multimodal and, overall, more complex the post‐conflict sequence
**Audience and external effects hypothesis (AEH)**. Social audience and environmental context can influence signaling strategies during post‐conflict interactions.	**AE1—Social audience size**. The presence of neighboring units and more adult bystanders is expected to increase reconciliation likelihood to avoid social consequences.	**Model 1** (latency/probability of reconciliation)	**Supported**
	**AE2—Third‐party support**. Agonistic support provided by bystanders during aggression is expected to reduce the need for direct reconciliation between opponents.	**Model 2** (probability of reconciliation)	**Not supported**
	**AE3—Anthropogenic disturbance**. The presence of humans may negatively interfere with natural conflict−management strategies.	**Model 1** (latency/probability of reconciliation)	**Not supported**

*Note*: The table also provides a summary of the outcomes, identifying the Bayesian model(s) supporting each prediction in cases of partial or variable support across response variables.

Treating these pillars as integrated lenses rather than independent factors, our framework uses a species whose communicative repertoire likely evolved under complex sociality and aligns with broader models of communicative and behavioral flexibility based on the integration of past experience and immediate cues to reduce uncertainty and guide adaptive behavior [22, 36].

## 2 Methods

### 2.1 Study Groups, Data Collection, and Coding

Data were collected in 2024 in the unprotected area of Debre Libanos (central highlands of Ethiopia, 9.711944°N, 38.8475°E, Oromia Region), where 17 one‐male units (OMUs) habituated to the presence of researchers [72] and with most adult individuals identified (*n* = 133), were followed from (8AM−4PM) as they daily visited two grazing areas (Sett Debre, Site 1, February−April, 10 OMUs; Godo, Site 2, October−December, seven OMUs) (Figure S1). No individuals from Site 1 were re‐encountered at Site 2, a result corroborated by genetic analyses. Animals were categorized as adults (fully grown, clear sexual traits; females, chest and neck swellings as an additional indicator; [78]), subadults, or juveniles (underdeveloped individuals usually near mothers). Because birth dates were unknown, the distinction between subadults and juveniles may be somewhat imprecise. We conducted scan sampling at 15‐min intervals, recording the number and identity of OMUs present, and noting OMUs within 50 m of one another. This allowed us to have a measure for the observation time of the different OMUs (total of 598 h, 2389 scans, 1502 in February−April, 887 in October−December, Table S1 for details). We recorded (SONY Full HD FDR‐AX43A, Sennheiser MKE600 microphone) agonistic and grooming interactions with all‐occurrence and focal sampling methods [79, 80]. The concurrent presence of three to five observers allowed to maximize and balance the group units in the video recordings. Multiple redundant recording was avoided by constant communication among observers about groups monitored. The duration of each aggressive event was measured from the onset of the first agonistic behavior displayed by the aggressor to the final agonistic behavior exhibited (Table S2 for definitions of agonistic patterns). After the end of aggression, we conducted 3‐min (or less, if victims/aggressors could not be followed) post‐conflict (PC) focal observations on both opponents [80]. Each PC was paired with a matched‐control (MC) focal observation of the same duration [74], on both the previous opponents. The MC was chosen on the closest available day, at a time closely matching the PC (within 2.5 h apart); both opponents had to be within 5 m, awake, and not engaged in any social interactions [60]. The MC was performed no sooner than 3 min after any agonistic interaction within the group [80].

For each observation, we coded the identity, age class, and sex of victims and aggressors, the number of OMUs in proximity, the outcome of the interaction (categorized as unclear when involving retaliation, counterattacks, or absence of fleeing or submission by the victim), and the presence of agonistic support during the ongoing aggression. Agonistic support was coded when a third individual, not involved at the start of the aggression, directed agonistic behavior toward the victim (polarizing support) or toward the aggressor (leveling support) [81]. All cases in which the roles of victim and aggressor were ambiguous were excluded from analyses. We also coded the presence of distress vocalizations emitted by the victim during the aggression and the level of physical contact involved (0 = no physical contact; 1 = low‐intensity physical contact, such as brief or incidental bodily contact during the interaction, without any forceful actions; 2 = high‐intensity physical contact involving active aggressive behaviors such as slapping, pulling, or biting). During PCs and MCs, we coded all affiliative patterns and signals exchanged between the victim and the aggressor (Table S3), as well as instances of self‐scratching. We coded behavioral patterns described to be used by geladas during post‐conflict affiliation [29, 35, 68, 70, 82], including patterns of physical affiliation (grooming, gentle touch, genital contact, body contact), and communicative signals from different sensory modalities (i.e., vocalizations, facial expressions, body postures) (Table S3). We coded the type and timing of the affiliative signal/pattern, the identity of the initiator, and whether the recipient responded with affiliative pattern/signals. As reconciliation is defined as the first affiliative contact exchanged between former opponents [80], we focused on the first exchange of affiliative signal(s)/contact between the opponents. If a third‐party affiliation occurred before an affiliative contact, this was not considered conciliatory.

We coded signals as directed toward a specific recipient when at least one of the following criteria was met: the signaler's head/body was oriented toward the recipient; the signaler was approaching or was already in physical contact with the recipient [83]. Details on coding reliability in Supplementary Text.

Although the study was noninvasive, approval for the observational protocols used was obtained from the Bioethical Committee of the University of Pisa (OPBA, n. 14/2023). Research procedures strictly adhered to the approved guidelines of the Ethiopian Wildlife Conservation Authority.

### 2.2 Post‐Conflict Sequences

Although behavioral patterns involving tactile contact (e.g., allogrooming, mounting) are often not classified as components of communicative sequences—being typically interpreted as outcomes following prior signaling [9, 35, 84], we chose to include them in our analysis as we are interested in the complexity of the (first) conciliatory interaction, and tactile patterns have historically been recognized as a primary modality of conflict resolution [37, 42, 85]. Moreover, their clear directional nature makes them relevant for understanding conciliatory dynamics. We defined a post‐conflict sequence as the first series of signals and behavioral patterns produced by the same signaler toward a single former opponent during the first PC interaction. We considered as post‐conflict sequence the one produced by the individual initiating signaling [9]. This sequence could include visual and acoustic signals (e.g., facial expressions, vocalizations), and affiliative physical patterns (e.g., grooming, mounting), as well as their combinations. For patterns to be part of the same sequence, they had to be produced in a continuous flow, with no more than 2 s between them [12, 86]. In addition, a sequence was terminated if it was interrupted by (1) any interaction with third‐party individuals or (2) the resumption of grazing activity. To assess the complexity of signaling sequences, we considered three main components: the duration of the sequence (seconds), the number of modalities involved (ranging from one to three across visual, tactile, and vocal), and a weighted sum (see below) reflecting the variety and intensity of the different behavioral patterns used. Following a perceptual‐based definition of multimodality [14, 87], we considered signals as multimodal when they were conveyed through different sensory modalities—visual, acoustic, and tactile channels.

When calculating the sequence duration, we excluded the duration of grooming bouts or spent in body contact—which consistently occurred at the end of the sequence—to avoid inflating duration. This was necessary as these patterns, once initiated, can persist for several minutes and would introduce variability unrelated to the structure of the initial signaling sequence. Such patterns were still considered to calculate the other metrics (number of signal/pattern types, multimodality). Cases with post‐conflict sequences composed solely of grooming (*n* = 6) or body contact (*n* = 2) were excluded from the analyses for parsimony, as they lacked actual communicative signals in their structure. Nevertheless, we acknowledge the need of conducting more research on the possible communicative nature of grooming in relation to the duration of the bouts.

We then calculated, for each sequence, a composite complexity index by summing the number of modalities used to the weighted sum of types of patterns used and then multiplying the value by the sequence duration. To capture the qualitative dimension of these sequences, we assigned different weights to patterns according to their known social intensity. For vocal signals, we followed previous knowledge on gelada calls [29, 35], assigning a higher score [2] to complex vocal sequences (including gelada‐derived vocalizations, moans, and/or wobbles), and a lower score [1] to simpler vocalizations such as grunt‐only or pre‐copulation calls. A similar scoring was applied to behaviors involving tactile contact, based on both the degree of physical engagement and the associated energetic investment. Specifically, grooming, social play, and mounting were scored as 2, as they involve sustained contact and active participation, whereas genital sniffing, gentle touch, or sitting in contact were scored as 1, as they involve brief or low‐intensity contact with limited physical engagement. Such brief tactile interactions are known to differ from sustained affiliative behaviors in their neurophysiological effects and social functions in receivers [88]. All visual signals recorded—rear‐presenting, lip‐smacking, and bared‐teeth lip flipping—were parsimoniously considered equally intense and assigned a score of 1. This decision was made in the absence of empirical evidence indicating differences in production costs or energetic investment among these display types. The cumulative score reflected both the number of distinct patterns and sensory modalities used (in case of combinations), as well as their quality and duration. While we acknowledge the partial availability of direct supporting literature, we are confident that our criteria provide a consistent framework to capture the variability in post‐conflict sequences. The index integrates weighted intensity of signals, modalities involved, and sequence duration—offering an informative measure of communicative complexity.

### 2.3 Genetic analyses for kinship

DNA from fecal samples (*n* = 102 subjects successfully genotyped) was genotyped using 16 microsatellite loci (Table S4). Kinship was inferred using ML‐Relate, COLONY 2.0.7.1, and CERVUS 3.0. COLONY was prioritized for final assignments due to its full‐pedigree likelihood approach, with ML‐Relate and CERVUS used for cross‐validation. Dyads were classified as full/half siblings, parent−offspring, or unrelated. As aggression mostly involved unrelated and half‐sibling dyads, individuals were grouped as Unrelated or Kin (sharing at least one parent). Full methodological details provided in the Supplementary Text in the Supplementary Material.

### 2.4 Dominance Hierarchy and Social Bond

We used the average dominance index [89] to derive intragroup‐unit ranking scores, and we quantified social bond strength using a standardized and derived version of the composite sociality index based on grooming interactions. Details provided in the Supplementary Material.

### 2.5 Statistics

#### 2.5.1 Bayesian Modeling

We conducted analyses within a Bayesian framework. Full details on models are provided in the Supplementary Text. Table 1 summarizes how hypotheses and predictions are tested in the models.

#### 2.5.2 Part A. The Presence and Modulation of Post‐Conflict Sequences

Model 1 tested if the emission of signals or physical affiliation were more likely and faster during PC versus MC. Focusing on PC observations, we then explored predictors of post‐conflict affiliation (Model 2).

**Time to signal or affiliative pattern emission between former opponents**. **Model 1a** (*n* = 610 PC‐MC observations). To investigate the factors influencing the production of post‐conflict sequences between former opponents, we analyzed the latency to the first affiliative pattern/signal (emitted either by the aggressor or the victim) using a Bayesian time‐to‐event modeling framework. For each PC and matched control event, we measured the time (in seconds) from the beginning of the observation period until the first affiliative interaction between the former opponents. When no such interaction occurred within the observation window (maximum 180 s), the observation was treated as right‐censored. Latency to reconciliation was modeled using a Bayesian survival model implemented in the R package *brms*. We specified a Weibull distribution for the time‐to‐event response, which allows flexible modeling of hazard rates over time. Fixed effects: *Condition* (PC/MC), *Age* (adult/subadult/juvenile), *Sex* (f/m) of aggressor and victim, *Group unit size* (number of adults in the OMU), number of adult *Subjects in proximity*, level of *Anthropogenic impact* during aggression (humans and livestock, only humans, only livestock, no disturbance). In this framework, positive coefficients indicate an increased hazard of affiliative contact (i.e., faster production of signals). Random intercepts: *Day*, *Group unit*, *Victim* and *Aggressor* identity, and the *Victim*:*Aggressor* dyad.

**Time to visual signals versus vocal signals versus physical affiliation between former opponents**. **Models 1b−d** (*n* = 6 observations in all models). We then analyzed the latency to the first signal exchanged or physical affiliation between former opponents during PC and MC periods to see if all forms of first signals (acoustic, tactile, visual) are more likely to occur earlier after conflicts. The first signal emitted was treated as the focal conciliatory act. We fitted three separate Bayesian time‐to‐event (survival) models corresponding to the first occurrence of visual signals (Model 1b), vocal signals (Model 1c), and physical affiliation (Model 1d). All three models had the same fixed and random effects of Model 1a.

Model 1 tests Predictions AE1—Social audience size, AE3—Anthropogenic disturbance, RV2—Sex combination (Table 1).

**The role of nonphysical signals in leading to physical affiliation**. While in the present work we include tactile signals/physical affiliation as part of post‐conflict sequences, here we explicitly separate the initial communicative act (visual or vocal signals) from physical affiliation to test whether such signals may act as ice‐breakers (physical affiliation was either preceded or not preceded by vocal or visual signals, but we never observed the opposite pattern). We modeled the occurrence of physical affiliation (yes/no) following the first signal using a Bayesian Bernoulli mixed‐effects model with a logit link function. The predictor was the presence of nonphysical signals exchanged (vocal, visual, or none). Random intercepts: Victim*Aggressor identity interaction.

**The factors affecting the presence of post‐conflict sequences**. **Model 2a** (*n* = 255 PC observations where all variables could be coded). The response variable (the occurrence of post‐conflict sequences in PCs) was modeled using a Bernoulli distribution with a logit‐link function. Fixed effects: *Aggression duration*, presence of *Agonistic support* during the aggression (no support, polarizing support, leveling support), *Victim scream duration* and *Physical contact* (0/1/2) during the aggression, *Social bond* between opponents, aggressor−victim *Rank difference*, *Sex* and *Age class* of both subjects. Aggression involving juveniles as well as those for which not all variables were codable were excluded; Random intercepts: *Day*, *Group unit*, *Victim* and *Aggressor* identity. Model 2b included the aggressor−victim *Kin relationship* (Unrelated/Kin dyads) on the subset of data for which the measure was available (Methods) (leading to *n* = 175 observations).

Model 2 tests Predictions RV1*—*Social bond strength, RV2*—*Sex combination, RU1*—*Kinship, RU2*—*Rank proximity, RU3*—*Aggression intensity, EM1*—*Victim distress, EM2*—*Dominance asymmetry, AE2*—*Third‐party support (Table 1).

#### 2.5.3 Part B. Complexity and Flexibility in Post‐Conflict Sequences

We examined predictors affecting the complexity of post‐conflict sequences, including its duration (Model 3) and uni‐ versus multimodality (Model 4). We also modeled the overall complexity of the sequence (*complexity index*) including duration, number of sensory modalities, and number of different signal types used (Model 5). These analyses regard reconciled conflicts (*n* = 151 total cases) in which all the variables (e.g., sequence features, social bond) could be coded (*n* = 126 cases). Each model has a *b* version on a subset of data on which kin relationship was available (*n* = 82 cases).

**The length of post‐conflict sequences**. **Model 3a** (*n* = 126 observations where all variables could be coded). The Bayesian model (gamma distribution, log‐link function) investigates factors affecting the length of the post‐conflict sequence. Fixed effects: *Aggression duration*, *Victim scream duration*, *Physical contact* during the conflict, *Social bond* between opponents, aggressor−victim *Rank difference*, *Role of the signaler* during the aggression (aggressor, victim), *Sex of the signaler, Sex of the receiver, Sex combination a‐v* (two levels: male aggressor, female victim: MF and same‐sex contestants, SS; this could either be female aggressor and victim, or male aggressor and victim), as well as the interactions Social bond*Sex combination and Rank difference*Sex combination. Importantly, we introduced the interactions, as the effects of rank and social bond strength on reconciliation outcomes may differ for female−female and male−female dyads, especially due to the alpha male dominance over the females and to the fact that a good portion of our aggressive events concerned male aggressors to female victims. This helps us disentangle sex effects due to different roles of males and females from the possible influence of dominance power or affinitive relationships. Random effects included the *Group unit*, as well as *Victim* and *Aggressor* identity. Model 3b (*n* = 82 observations where all variables could be coded) included the *Kin relationship* between the aggressor and the victim. The model was simplified as much as possible in its structure to avoid overfitting due to the lower number of observations. We decided to keep predictors likely affecting the response variable (after running the version *a* of the model) but also those important to be controlled of while testing the effect of familiarity (sex, social bond, rank); thus, fixed factors included the *Kin relationship* between the two subjects (unrelated/half or full siblings), the *Social bond* between opponents, aggressor−victim *Rank difference*, the *Sex of the signaler* and *of the receiver*, and *Victim scream duration*. Random effects included the *Victim* and *Aggressor* identity.

Model 3 tests Predictions RV1*—*Social bond strength, RV2*—*Sex combination, RU1*—*Kinship, RU2*—*Rank proximity, RU3*—*Aggression intensity, EM1*—*Victim distress, EM2*—*Dominance asymmetry (Table 1).

**The uni‐ or multimodal nature of post‐conflict sequences**. **Model 4a** (*n* = 126 observations). The model (Bernoulli distribution, log‐link function) investigates the factors affecting the modalities (one vs. multiple) used in the sequence. The model structure (fixed and random effects) was as in Model 3a. In Model 4b (*n* = 82 observations), we added the *Kin relationship* and simplified the model as in Model 3b.

Model 4 tests Predictions RV1*—*Social bond strength, RV2*—*Sex combination, RU1*—*Kinship, RU2*—*Rank proximity, RU3*—*Aggression intensity, EM1*—*Victim distress, EM2*—*Dominance asymmetry (Table 1).

**The complexity index of post‐conflict sequences. Model 5a**. The model (gamma distribution, log‐link function) investigates the overall complexity of post‐conflict sequences (response variable: *complexity index*). Model structure was equal to Model 4a. In Model 5b, *Kin relationship* was added, and the model was simplified to avoid overfitting by excluding Model 5a factors whose posterior distribution indicated no effect (*Aggression duration, Physical contact*, *Role of signaler*) and simplifying random structure (*Victim* and *Aggressor* identity).

Model 5 tests Predictions RV1*—*Social bond strength, RV2*—*Sex combination, RU1*—*Kinship, RU2*—*Rank proximity, RU3*—*Aggression intensity, EM1*—*Victim distress, EM2*—*Dominance asymmetry (Table 1).

## 3 Results

### 3.1 Descriptive Results

We recorded 3035 grooming and 508 intragroup agonistic interactions, among which ⁠305 were overt aggression (direct, physically expressed aggressive behavior directed toward a conspecific, excluding cases with only threat displays or avoidance behaviors) paired with 305 MC observations. These interactions involved 76 aggressors and 114 victims (197 unique aggressor−victim dyads). Males were aggressors in 180 cases (127 cases over a female, 53 cases over another male), females in 130 cases (107 cases over another female, 23 cases over a male). Mostly adults were involved in aggression (the aggressor was adult in 275, subadult in 31, and juvenile in 4 cases; the victim was adult in 223, subadult in 51, and juvenile in 36 cases). In 151 post‐conflicts and 19 MCs, affiliative signals and/or behavioral patterns were produced by one or both opponents; in 121 out of 151 cases, the aggressor started the contact, and in 64 out of 151 interactions, affiliative signals and/or behaviors were reciprocated (both subjects emitting signals). Geladas emitted either vocal (grunts, derived affiliative sequences, pre‐copulation calls), visual (rear presenting, lip‐smacking, bared‐teeth lip flip), or tactile (allogrooming, genital sniffing, mounting, gentle touch, social play) signals/patterns composing their post‐conflict sequences (Tables S5 and S6). Sequence durations ranged from 0.6 to 20.4 s (excluding grooming interactions). Post‐conflict sequences in geladas were mostly unimodal, primarily vocal (*n* = 120) or visual (*n* = 37). Derived vocal sequences (*n* = 77) and grunts (*n* = 42) were most common, followed by rear presenting (*n* = 17) and lip‐smacking (*n* = 12). However, 51 sequences combined multiple modalities, and 55 included more than one signal/behavior type.

### 3.2 Part A. The Presence and Modulation of Reconciliation

#### 3.2.1 Model 1a

**FIGURE 2:**
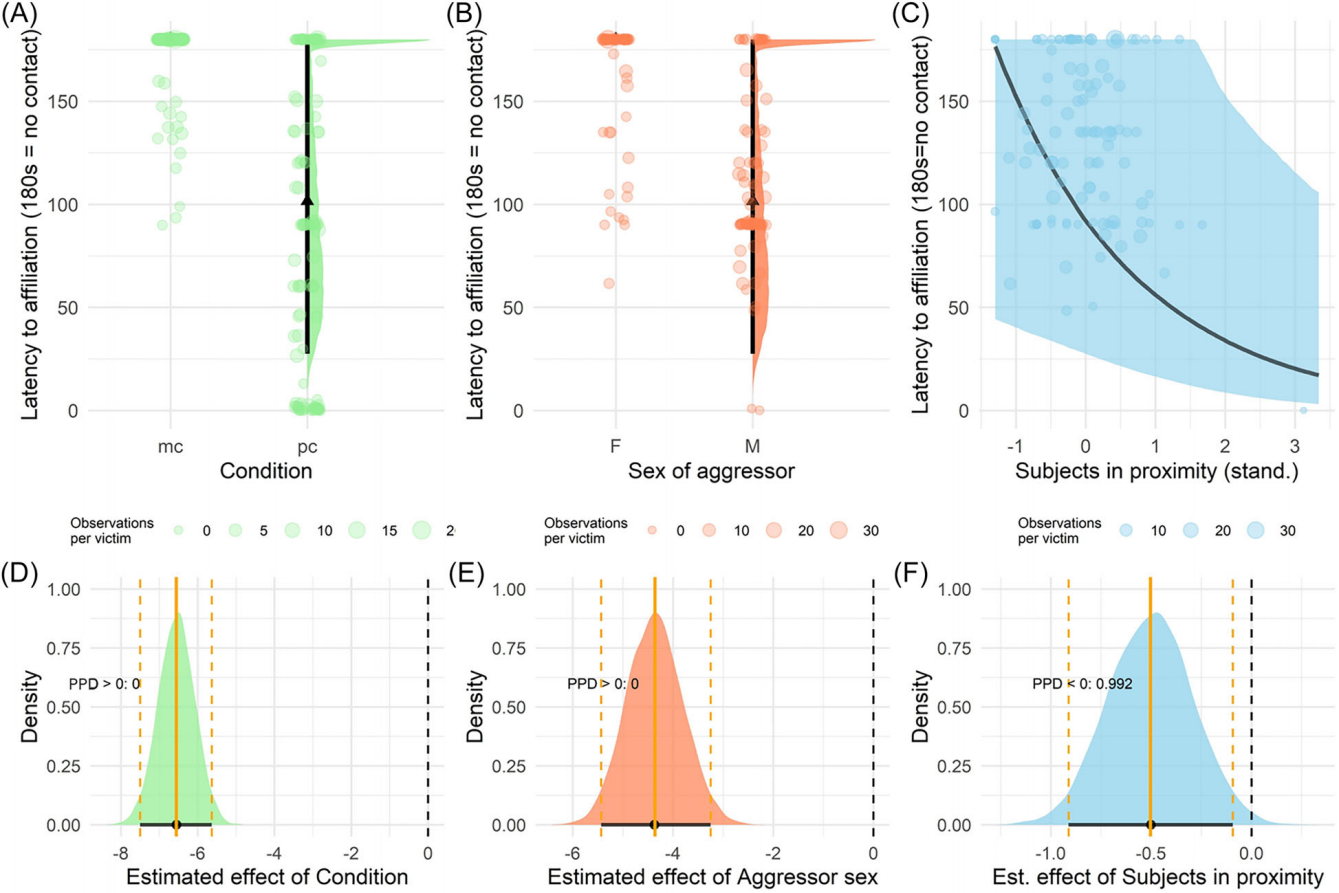
Latency to the production of post‐conflict sequences as estimated from Model 1. Latency (in seconds) was modeled using a Bayesian Weibull survival model with right‐censoring at 180 s (i.e., no post‐conflict sequence within the observation window). Panels (A−C) show model‐predicted latency according to (A) condition, (B) sex of the aggressor, and (C) number of subjects in proximity (scaled). Shaded half‐eye densities represent posterior distributions of predicted latency values; black triangles and horizontal bars indicate posterior means and 95% credible intervals. Colored points represent observed victim‐ or aggressor‐level mean latencies, with point size proportional to the number of interactions recorded for each subject. Panels (D−F) show posterior distributions of the estimated effects of (D) condition, (E) sex of the aggressor, and (F) number of subjects in proximity. Solid orange lines indicate posterior means and dashed orange lines the 95% credible intervals. The dashed black line marks the zero‐reference value; annotations report the posterior probability that the effect is greater than zero (PPD > 0).

**The exchange of post‐conflict signals and patterns in PC versus MC observations** (*n* = 610 PC and MC observations). There was strong evidence that nonaggressive signals were exchanged earlier in PC than in MC observations (estimate = −6.55, 95% CI [−7.50, −5.63], *p* ^−^ = 100%) (Figure 2A,D). The latency was also shorter when the aggressor was male (estimate = −4.36, 95% CI [−5.43, −3.25], *p* ^−^ = 100%) (Figure 2B,E). Conversely, reconciliation tended to occur later when the aggressor was a subadult (estimate = 1.84, 95% CI [0.32, 3.37], *p* ^+^ = 99.1%). The number of adult individuals in proximity was negatively associated with latency (estimate = −0.50, 95% CI [−0.91, −0.09], *p* ^+^ = 99.2%) (Figure 2C,F), suggesting that sequences were produced sooner when more adults were nearby. In contrast, there was little evidence that the age class of the victim, OMU adult group size, or the level of anthropogenic disturbance during the aggression influenced reconciliation (Table S7). See also Table 1 for how these outcomes relate to hypotheses and predictions.

#### 3.2.2 Models 1b−d

**Time to visual signals versus vocal signals versus physical affiliation between former opponents** (*n* = 620 observations). Across all modalities, there was strong evidence that the first signal was produced earlier in PC than in MC observations (visual: estimate = −6.55, 95% CI [−7.48, −5.64]; vocal: −6.55 [−7.50, −5.64]; tactile: −6.56 [−7.48, −5.65]; *p* ^−^ = 100% in all models; Tables S8–S10). Across signal types, the latency to signaling was consistently shorter when the aggressor was male (visual: −4.36 [−5.43, −3.26]; vocal: −4.37 [−5.48, −3.26]; tactile: −4.36 [−5.45, −3.24]; *p* ^−^ = 100%), and longer when the aggressor was a subadult (≈1.84 across models; *p* ^+^≈99%). Increased adult proximity reduced latency across all modalities (≈−0.50 across models; *p* ^+^≈99%). Other predictors showed weak or uncertain effects (Tables S8–S10).

Model 1 supports Predictions RV2—Sex combination and AE1—Social audience size, no support for AE3—Anthropogenic disturbance (Table 1).

#### 3.2.3 The Role of Nonphysical Signals in Leading to Physical Affiliation

While the presence of nonphysical signals was associated with a higher probability of subsequent physical affiliation (β = 0.67, 95% CrI [−0.91, 2.34]), credible intervals overlapped zero, indicating substantial uncertainty in the effect. In more detail, while physical affiliation was always preceded by nonphysical signals when it occurred, many instances of visual and vocal signals were not followed by affiliative contact. This asymmetry likely contributes to the observed uncertainty in the effect and explains the wide credible intervals.

#### 3.2.4 The Factors Affecting the Presence of Post‐Conflict Sequences

**Model 2a** (*n* = 255 PC observations where all variables could be coded). The production of post‐conflict sequences was higher with male aggressors (estimate = 6.12, 95% CI [3.20, 11.76], *p* ^+^ = 100%) and lower with subadults (estimate = −3.61, 95% CI [−9.08, −0.14], *p* ^−^ = 98%) (Table S11). **Model 2b** (*n* = 175). Considering the subset of observations for which we had genetic data about victims and aggressors, PC affiliation probability was still higher with male aggressors (estimate = 4.04, 95% CI [0.06, 1.62], *p* ^+^ = 100%) but also after longer victim screams (estimate = 0.79, 95% CI [0.19, 3.09], *p* ^+^ = 98.3%); only moderate support for longer aggression leading to lower probability of reconciliation (estimate = −0.94, 95% CI [−2.09, −0.02], *p* ^−^ = 97.6%) was found. The effect of *Kin relationship* was inconclusive (Table S11). See also Table 1 for how these outcomes relate to hypotheses and predictions.

Model 2 supports Predictions RV2*—*Sex combination and AE1*—*Social audience size, while no support for Prediction RV1*—*Social bond strength, RU1*—*Kinship, RU2*—*Rank proximity, RU3*—*Aggression intensity, EM1*—*Victim distress, EM2*—*Dominance asymmetry and AE2*—*Third‐party support (Table 1).

### 3.3 Part B. Complexity and Flexibility in Post‐Conflict Sequences

#### 3.3.1 The Length of Conciliatory Sequences

**FIGURE 3:**
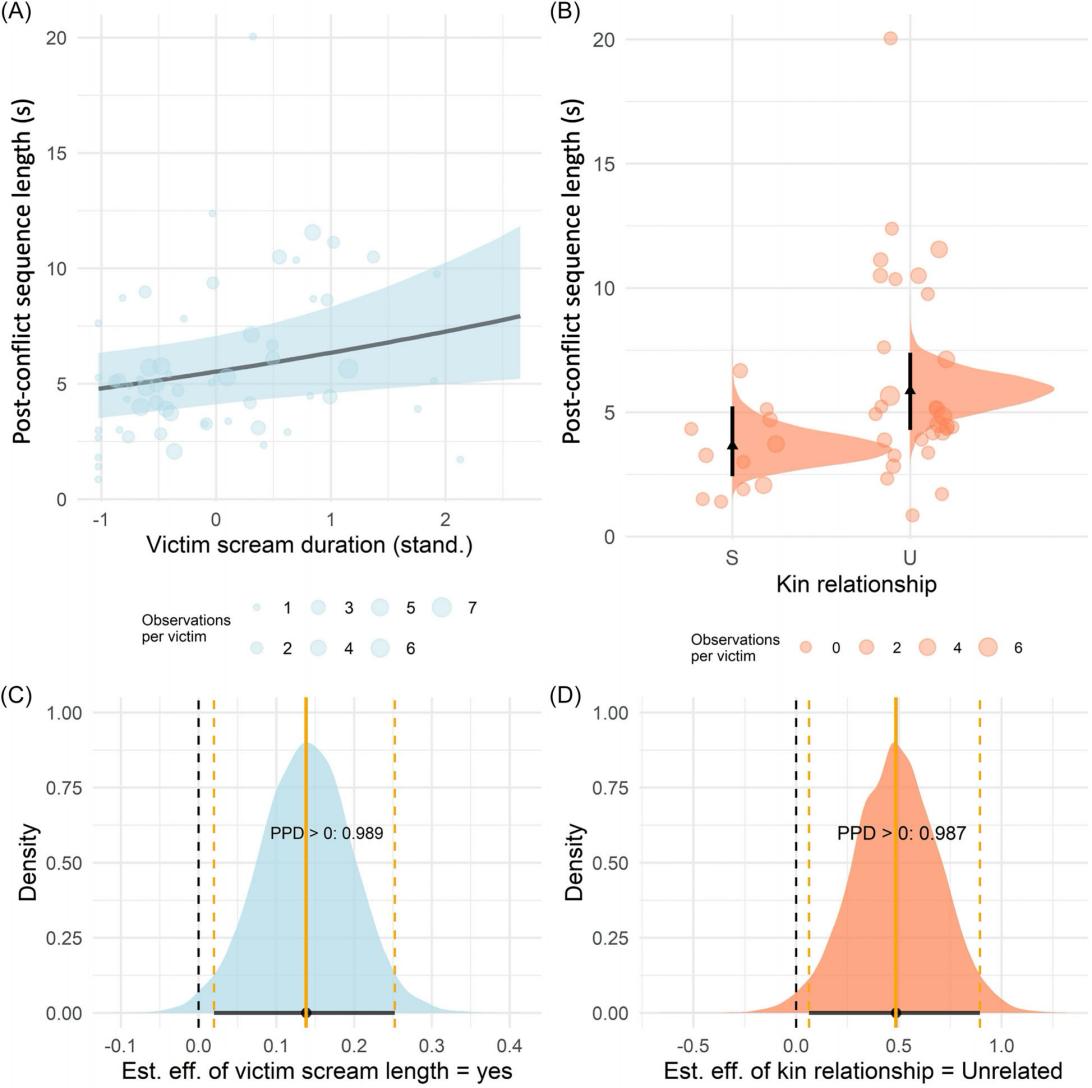
Length of post‐conflict sequences following aggressive interactions. Panels (A, B) show model‐predicted sequence length as a function of (A) victim scream duration (standardized) and (B) kin relationship between opponents (siblings/half‐siblings vs. unrelated), estimated from Bayesian regression models. In panel (A), the solid line represents the posterior mean prediction and the shaded ribbon the 95% credible interval. In panel (B), shaded half‐eye densities represent posterior distributions of predicted values, with black triangles and horizontal bars indicating posterior means and 95% credible intervals. Colored points represent observed victim‐level mean sequence lengths, with point size proportional to the number of interactions recorded per individual. Panels (C, D) show posterior distributions of the estimated effects of victim scream duration and kin relationship. Solid orange lines indicate posterior means and dashed orange lines the 95% credible intervals. The dashed black line marks the zero‐reference value; annotations indicate the posterior probability that the effect is greater than zero (PPD > 0).

**Model 3a** (*n* = 126 observations). The duration of victim scream had a positive effect on the length of the post‐conflict sequence (estimate = 0.14, 95% CI [0.02, 0.25], *p* ^+^ = 98.9%) (Figure 3A,C and Table S12). **Model 3b** (*n* = 82). Unrelated subjects exchanged longer sequences (estimate = 0.49, 95% CI [0.06, 0.89], *p* ^−^ = 98.7%) (Figure 3B,D). Again, longer victim screams led to longer post‐conflict sequences (estimate = 0.14, 95% CI [0.00, 0.28], *p* ^+^ = 97.8%) as for higher aggressor−victim rank distances (estimate = 0.18, 95% CI [0.01, 0.34], *p* ^+^ = 96.9%) (Table S12 for full results). See also Table 1 for how these outcomes relate to hypotheses and predictions.

Model 3 supports Predictions RU1*—*Kinship, EM1*—*Victim distress, while no support for Prediction RV1*—*Social bond strength, RV2*—*Sex combination, RU2*—*Rank proximity, RU3*—*Aggression intensity, EM2*—*Dominance asymmetry (Table 1).

#### 3.3.2 The Uni‐ or Multimodal Nature of Post‐Conflict Sequences

**FIGURE 4:**
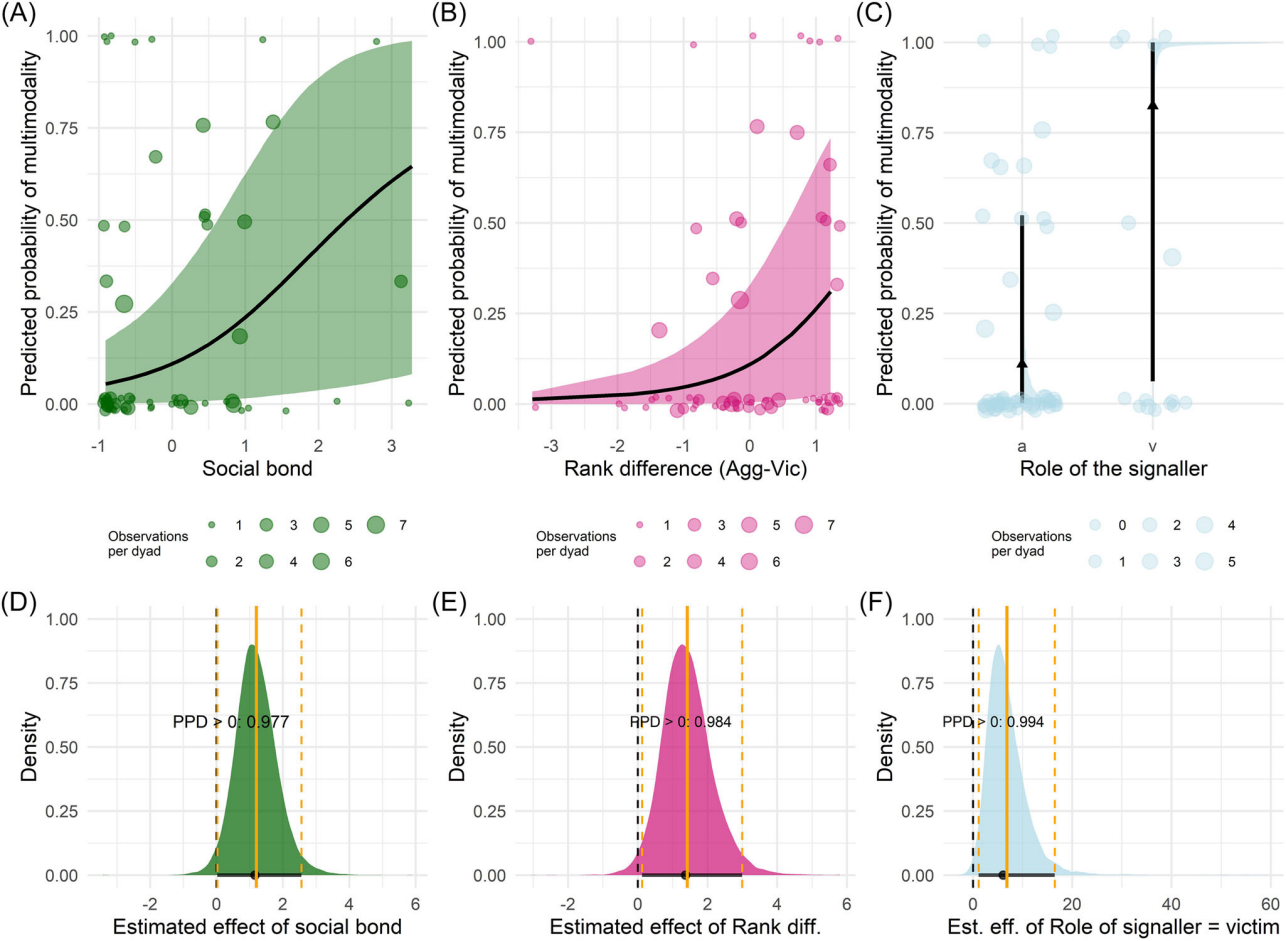
Probability that post‐conflict sequences are produced in a multimodal form following aggressive interactions. Panels (A−C) show model‐predicted probabilities according to (A) social bond strength between opponents, (B) aggressor−victim rank difference, and (C) the role of the signaler (aggressor vs. victim). Solid lines represent posterior mean predictions and shaded ribbons the 95% credible intervals. Points represent observed dyad‐level mean probabilities, with point size proportional to the number of interactions recorded per dyad. Panels (D−F) show posterior distributions of the estimated effects of social bond, rank difference, and signaler role. Solid orange lines indicate posterior means and dashed orange lines the 95% credible intervals. The dashed black line indicates the zero‐reference value; annotations report the posterior probability that the effect is greater than zero (PPD > 0).

**Model 4a** (*n* = 126). Higher aggressor−victim rank differences were associated with an increased probability of using multimodal rather than unimodal signals (estimate = 1.42, 95% CI [0.1, 2.99], *p^+^* = 98.4%) (Figure 4A,D). Victims emitting post‐conflict sequences had a higher likelihood of using multimodal signals compared to aggressors (estimate = 6.84, 95% CI [1.13, 16.47], *p^+^* = 99.3%) (Figure 4B,E). Moreover, dyads sharing stronger social bonds generally produced more multimodal sequences (estimate = 1.20, 95% CI [0.03, 2.56], *p^+^* = 97.7%) (Figure 4C,F). By contrast, longer victim screams showed a negative association with multimodal signaling (estimate = −1.15, 95% CI [−2.38, −0.16], *p^−^* = 98.9%) (Table S13). **Model 4b** (*n* = 82 observations). When focusing on dyads for which genetic data could be extracted, rank difference and victim scream duration similarly affected the use of multimodal signals, while the effect of Kin relationship was inconclusive (Table S13). See also Table 1 for how these outcomes relate to hypotheses and predictions.

Model 4 supports Predictions RV1—Social bond strength and EM2—Dominance asymmetry, while no support for Prediction RV2—Sex combination, RU1—Kinship, RU2—Rank proximity, RU3—Aggression intensity, EM1—Victim distress (Table 1).

#### 3.3.3 The Complexity Index of Post‐Conflict Sequences

**Model 5a** (*n* = 126). Increasingly dominant aggressors over victims were associated with increased signal complexity in the sequences (*complexity index*) (estimate = 0.27, 95% CI [0.05, 0.49], *p^+^* = 99.2%). There was also a significant positive effect of social bond strength (estimate = 0.20, 95% CI [0.00, 0.39], *p^+^* = 97.6%) (Table S14). **Model 5b** (*n* = 82). The sequences *complexity index* was higher for unrelated dyads compared to those sharing one parent (estimate = 0.51, 95% CI [0.03, 0.98], *p^−^* = 97.5%). Again, rank difference positively predicted complexity (estimate = 0.28, 95% CI [0.08, 0.47], *p^+^* = 99.7%) (Table S14). See also Table 1 for how these outcomes relate to hypotheses and predictions.

Model 5 supports Predictions RV1—Social bond strength and EM2—Dominance asymmetry, while no support for Prediction RV2—Sex combination, RU1—Kinship, RU2—Rank proximity, RU3—Aggression intensity, EM1—Victim distress (Table 1).

## 4 Discussion

**FIGURE 5:**
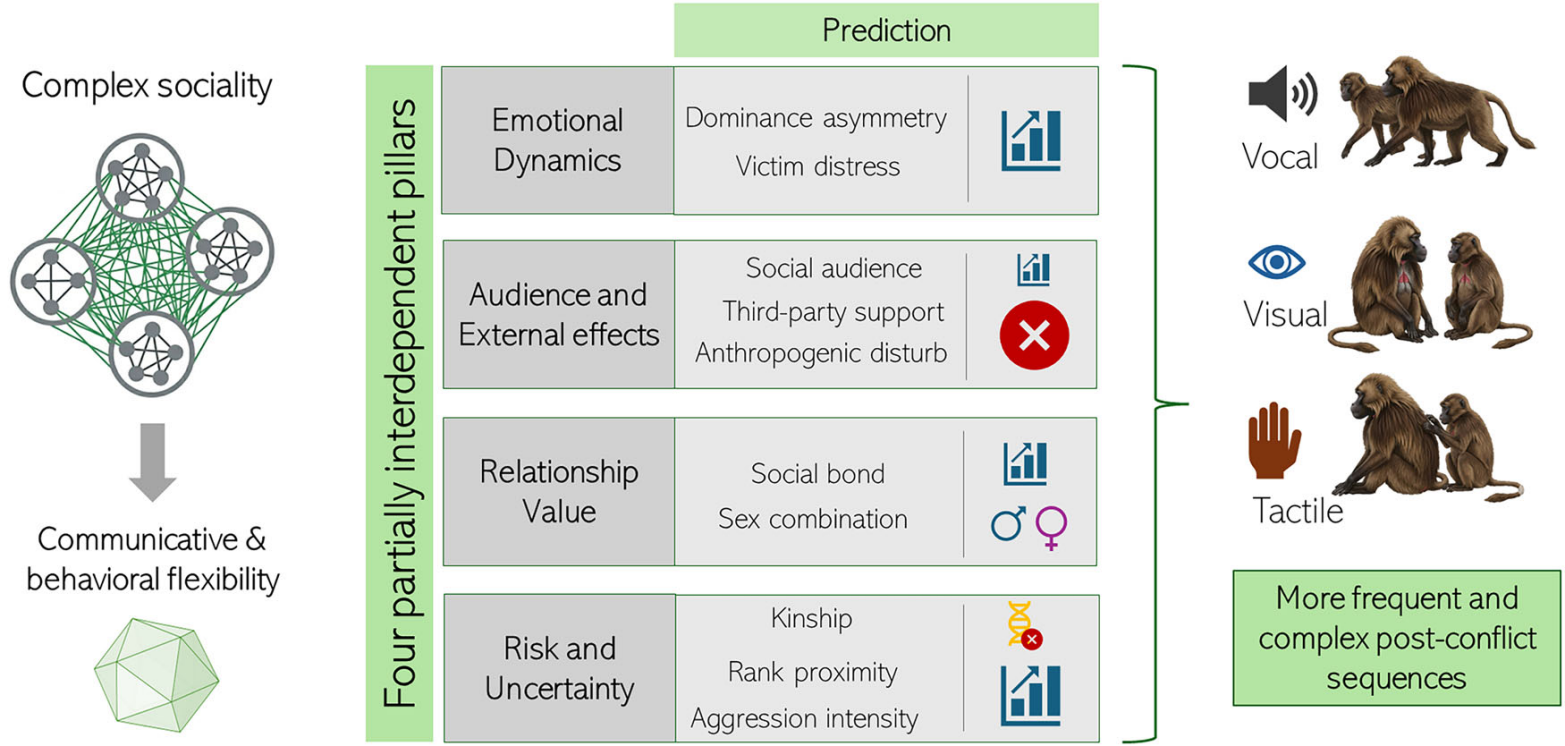
Conceptual framework illustrating the four proposed pillars (relationship value, emotional dynamics, risk and uncertainty, audience and external factors) shaping flexibility in communicative efforts in post‐conflict interactions under the pressures of social complexity. On the right of each variable included in the modeling framework, an icon indicates the condition or value under which that variable is predicted to positively affect the presence and complexity of conciliatory sequences in geladas. Importantly, the icons next to the variable names represent the variable values under which the presence and complexity of post‐conflict sequences is predicted to increase (upward arrow and bar chart = with increasing dominance asymmetry, victim distress, social audience, social bond, rank proximity, and aggression intensity should positively affect the response variable; red X = the absence of third‐party support and kinship should positively affect the response variable, male−female icon = mixed‐sex dyads should have highest rates and complexity in reconciliation). See Table 1 for a complete overview of the hypothesis framework and for a detailed explanation of which predictions have or have not been supported by the Bayesian model results.

Group living inevitably creates unpredictability, as individuals must interact with multiple partners who differ in rank, kinship, internal state, and relationship history [9, 17, 19, 22, 35]. Navigating this complexity, and the conflicts inevitably deriving from it, requires the sociocognitive skills to predict outcomes, grounded in memory of past encounters and in the rapid integration of new cues. In the present work, we used post‐conflict interactions between aggressors and victims to investigate communicative and behavioral flexibility. Our results show that geladas, monkeys living in complex social systems, integrate experience with incoming information updates to flexibly regulate the signals and patterns used to reduce hostility and potentially reassure victims after a conflict. The occurrence and structure of post‐conflict sequences were influenced by interdependent factors mapping onto the four proposed pillars of relationship value, risk and interactional uncertainty, emotional dynamics, and audience and external effects (Table 1 and Figure 5).

First, we document an increased use of nonaggressive vocal−visual signals and physical contacts after conflicts between former opponents in geladas. This aligns with previous captive gelada observations [68, 69] but contrasts with a wild population where reconciliation was not found [70]. Our larger sample and systematic video plus directional‐audio recordings likely allowed detection of all signals, especially vocalizations, which were the most frequent—probably because they enable addressing a former opponent with low energetic cost and minimal risk [45]. The recurrent production of calls is in line with literature on monkeys showing a prevalence of nonphysical vocal signals after conflicts [43, 46, 47]. While reconciliation typically occurs within minutes after a conflict [38, 39, 42, 68, 69], here it was almost always initiated within seconds, with aggressors often directing vocal sequences toward victims immediately after the agonistic exchange. Such rapid resolution may allow individuals to quickly restore social stability and resume grazing, a predominant activity for the species [90]. While signals exchanged may have a conciliatory function and be used to repair relationships, an alternative but not mutually exclusive interpretation is that, since aggression generates uncertainty about future interactions, post‐conflict sequences (especially vocal−visual signals) may function as indicators of nonaggressive intent that facilitate the resumption of social interactions. These perspectives are closely related and may not be empirically separable in the present dataset. Vocal and visual signals may thus convey reliable information about the caller's peaceful intent and reassure victims that the conflict will not escalate further [7, 43, 44, 46, 47]. Post‐conflict sequences may, therefore, not have a strictly conciliatory function but instead reflect the animals’ willingness to re‐engage socially. Although not strongly supported here, this interpretation is consistent with experimental evidence from primate playback studies showing that vocalizations can influence subsequent affiliative behavior and modulate the likelihood of peaceful interactions following aggression [44, 47]. On an even more parsimonious interpretation, the exchange of vocal−visual signals and physical affiliation may simply reflect a greater motivation to interact with socially preferred partners, such as closely bonded individuals. However, the fact that all signal types occurred significantly earlier and more frequently in post‐conflict than in matched‐control observations (Models 1b−d) suggests that they are not merely byproducts of baseline interaction tendencies. Rather, they appear to be specifically recruited in the immediate aftermath of aggression, potentially signaling nonaggressive intent toward the former opponent.

The fitness value of a partner can shape the investment in efforts deployed in the post‐conflict interaction, either to repair the relationship or simply to signal positive intentions [91]. Here, the strength of the aggressor−victim bond predicted higher diversity of signal types and modalities in post‐conflict sequences (supporting Prediction RV1—Social bond strength, Table 1). When controlling for the sex combination of the contestants, this effect was valid for both male−female and same‐sex dyads, highlighting the need to preserve social bonds across individuals. Conflicts with male aggressors (the most usual case) were more likely to be followed by nonaggressive signals. Male aggressors were also the subjects producing post‐conflict sequences most often, possibly reflecting a higher need for males to maintain group stability (partial support to Prediction RV2—Sex combination, Table 1). Indeed, gelada males need to sustain enduring bonds with multiple ingroup females [35, 61], making cross‐sex relationships especially valuable [35]. Moreover, gelada males face greater complexity than females [17, 19], since they need to maintain bonds with multiple nonkin females, while ingroup females are generally kin and need to maintain fewer differentiated relationships compared to males [35, 60]. Male aggressors might thus produce more sequences as a strategy to preserve high‐value cross‐sex bonds [35].

Sensitivity to perceived risk and interactional uncertainty also shaped, to some extent, the exchange of signals. Unrelated aggressor−victim dyads exchanged longer and more differentiated sequences compared to dyads sharing some degree of relatedness (supporting Prediction RU1—Kinship). Conflicts may pose less of a threat to social bonds among kin than among nonkin, reducing the need for intensive investment to repair relationships [92]. This appears as a risk‐sensitive strategy [9], particularly for male−female dyads (generally unrelated) and among those females who share relatively lower kinship levels [60]. On the other hand, aggression intensity did not significantly affect the complexity of post‐conflict sequences (Prediction RU3—Aggression intensity not supported).

Importantly, we acknowledge that the variables composing the four pillars are not fully independent but may interact in partially interdependent ways. As a result, the factor−effect classification (Figure 5 and Table 1) provides a simplification that does not entirely reflect real‐world dynamics. For instance, given the correlation between kinship and rank proximity in matrilineal societies, the observed patterns of increased reconciliation complexity among nonkin and more distantly ranked individuals should be interpreted cautiously, as they may reflect overlapping effects of both variables rather than independent influences. Moreover, unrelated individuals may nonetheless experience low social uncertainty if they share strong affiliative bonds or are separated by substantial rank distance. Conversely, interactions involving weak social bonds may still generate heightened negative affect even in the absence of clear dominance asymmetries or distress signals. We partially addressed these confounds by controlling for sex combination in interaction with social bond strength and rank differences. This is particularly relevant in geladas, where sex can shape both dominance relationships and affiliative patterns. Moreover, given the species’ social structure—where females predominantly form strong bonds with kin—some of these interactions are likely constrained in practice, although exceptions exist, particularly in male−female associations. Importantly, we recognize that more complex interdependencies could be better captured in future work by incorporating higher‐order interactions in larger datasets.

Emotion dynamics can clearly have an impact on post‐conflict interactions. Signals produced can indeed be tailored according to the affective states of the subjects involved in the interaction, aided by emotion recognition [93]. Here, victim scream duration increased the post‐conflict sequence length (supporting Prediction EM1—Victim distress), independently of who started reconciling. Although data on emotional signaling and its role in eliciting nonhuman empathic‐like responses is limited, our finding aligns with evidence in which screams can attract third‐party aid, whether strategically or not [2, 74, 94]. Although reconciliation was more often initiated by aggressors, victim‐produced sequences were more likely to include signals from multiple modalities. This suggests that when aggressors do not make the first move, victims make greater efforts, possibly to restore an internal affective and relational equilibrium. Since multisensory signals can be more effective on receivers than unimodal signals [13, 14, 95], victims may combine modalities to more efficiently communicate a conciliatory intent. This also appeared among socially close partners and in interactions with more dominant aggressors, where geladas more often combined multiple modalities when reconciling. All this considered, although early work has documented rhythmic and melodic control in gelada vocal exchanges [71], further research is needed to assess affective and strategic modulation of gelada signals, particularly considering intracall variability.

Differences in dominance power influence social uncertainty and the emotional dynamics of interactions. Across primates, species‐specific patterns shape how dominance influences reconciliation [44, 52, 53]. In our study case, the more dominant the aggressor over the victim, the greater the post‐conflict sequence complexity (Prediction EM2—Dominance asymmetry supported; Prediction RU2—Rank proximity not supported). When aggressors held a strong dominance advantage over victims, for instance, leader males or dominant females, this (supported by our result on the effect of victim scream duration) may have possibly increased fear or social vulnerability for the victim, leading to greater efforts to mitigate the possible consequences of the conflict. While the effect of rank was independent from the signaler role during the aggression, this result supports the view that in tolerant societies, dominant individuals often need to communicate more to manage uncertain interactions and maintain affiliative networks through social means rather than relying solely on rank or force [28, 96]. This can be especially valid for gelada males who maintain group stability without coercion [35, 56].

Overall, these results point toward a comforting and reassuring role [26] of post‐conflict sequences—more often initiated by dominant aggressors toward emotionally distressed subordinate victims—and to a signal complexity that is attuned to both affective states and relationship value.

The social environment surrounding the interaction can also influence the strategies adopted to restore social equilibrium. The presence of more subjects around victims and aggressors (i.e., more group units) led to a higher likelihood of reconciliation (Prediction AE1—Social audience size supported). Given gelada socioecology—where multiple units forage in close proximity with high tolerance but ongoing male competition—failure to reconcile in the presence of many nearby groups may increase risks of aggression escalation, renewed attacks, or female transfers and extra‐group mating [65]. On the other hand, the intervention of third parties during the aggression did not affect the rate of reconciliation (Prediction AE2—Third‐party support not supported), as the presence and activities of humans (Prediction AE3—Anthropogenic disturbance not supported), this latter likely reflecting the habituation of this wild population inhabiting a human‐impacted area [97].

Overall, our study shows that geladas can flexibly use post‐conflict sequences in relation to contextual information, integrating cues about relationship value, social risk, and affect [5, 9, 22, 36]. The ability to combine past experience with immediate perceptual inputs [22, 98] to calibrate socially competent behavioral responses [99] likely derives from the challenges of social living [36]. Examining dyadic post‐conflict interactions provides a fine‐grained view of interactional, social, affective, and environmental factors shaping communicative complexity [22] and allowing animals to maintain group cohesion in spite of conflict. Our findings hint at the interplay of social, cognitive, and communicative complexity: social complexity creates unpredictable interactions, cognitive complexity enables prediction and integration, and communicative complexity provides the behavioral tools to manage relationships and sustain group cohesion [16, 18, 36]. Future research using multimodal approaches should further examine whether the variability observed in conflict−resolution sequences also reflects underlying semantic variation in the meanings conveyed by those sequences [22, 100]. Moreover, testing communicative flexibility in species living in socially complex systems—such as fission−fusion societies (e.g., chimpanzee, spider monkeys), cooperatively breeding species (e.g., marmosets), or species differing in exogamic patterns—will be crucial to understand how social structure shapes the emergence and diversification of communicative strategies [16, 18]. Applying this multifactorial framework across taxa will help identify the selective pressures shaping flexible signaling in primates and clarify its continuity with human communication.

## Author Contributions

Study conception: L.P., A.L., and E.P.; Data collection: L.P., A.G., M.F., A.Q., and S.A.G.; Project funds seeking: E.P., B.A.B., V.S., and G.P.; Field logistic support: S.A.G. and H.T.A.; Genetic analyses: A.M., M.V.R., and P.L.A.; Data analyses: L.P.; Writing: L.P., A.L., and E.P.; Writing (editing and approval): all authors.

## Conflicts of Interest

The authors declare that they have no conflicts of interest.

## Supporting information




**Supporting Information**: nyas70352‐sup‐0001‐SuppMat

## Data Availability

Data used for the analyses are provided as Supplementary Material.
