# Supplementary material for: Relationship Value, Emotion, and Uncertainty Shape Conflict−Resolution Communication in a Wild Primate
Source: Ann N Y Acad Sci. 2026 Jul 30;1562(1):e70352. doi: 10.1111/nyas.70352 (PMC13419923; doi:10.1111/nyas.70352)
Supplement: Supplementary file 1 — Supporting Information: nyas70352‐sup‐0001‐SuppMat [file NYAS-1562-0-s001.docx]

**
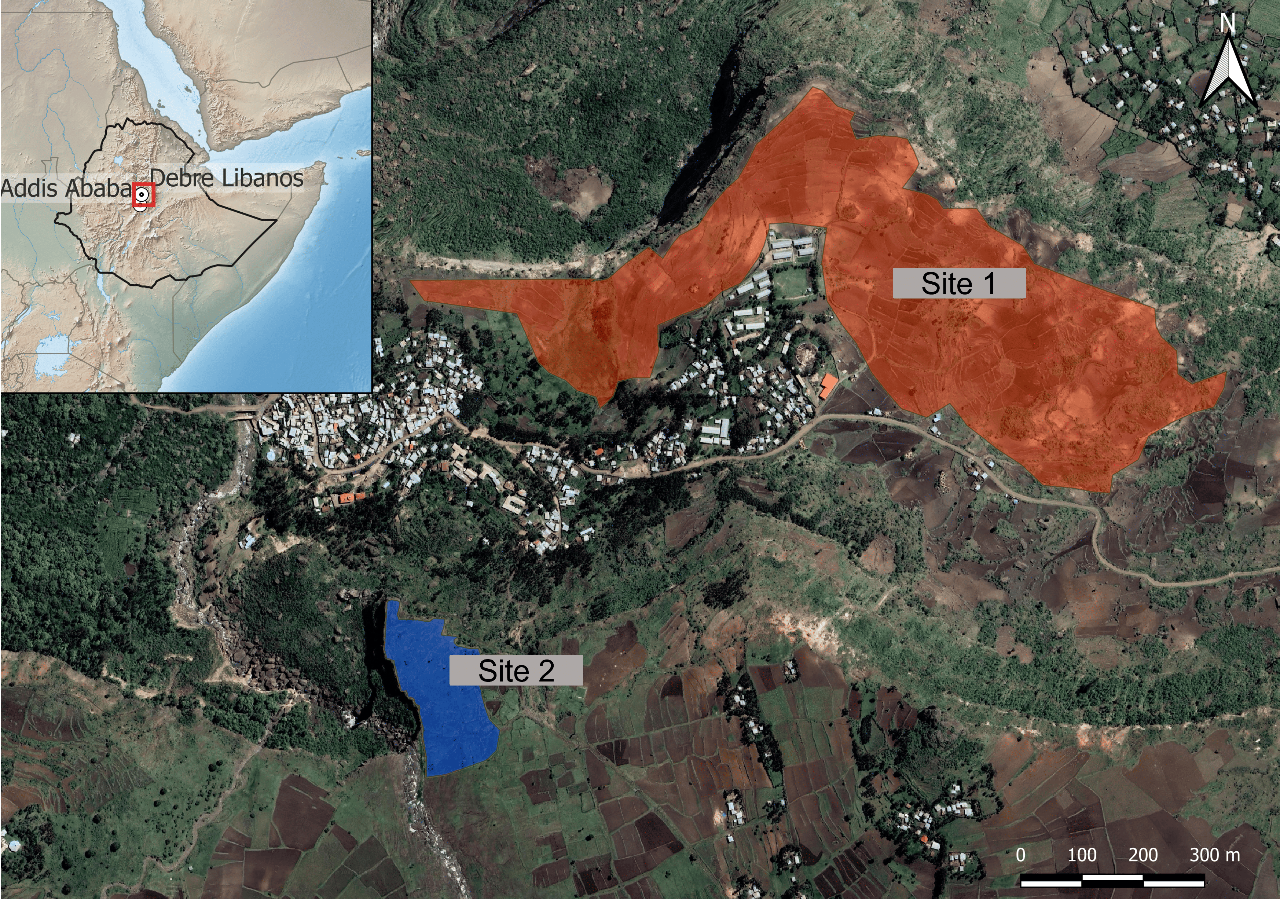
**

**Figure S1.** Map of the study sites in Debre Libanos (Ethiopia, 9.711944°N, 38.8475°E). Red and blue areas were generating connecting the GPS points recorded during scan-sampling.

| **OMU** | **SCAN** | **MINUTES** | **HOURS** | **SUBJECTS** | **SUBJECTS (no juvinf)** |
| --- | --- | --- | --- | --- | --- |
| **Paul** | 842 | 12630 | 210,5 | 22 | 15 |
| **Crown** | 693 | 10395 | 173,25 | 19 | 11 |
| **Small** | 667 | 10005 | 166,75 | 16 | 10 |
| **Medium** | 657 | 9855 | 164,25 | 17 | 11 |
| **Taylor** | 619 | 9285 | 154,75 | 10 | 7 |
| **Renato** | 532 | 7980 | 133 | 16 | 8 |
| **Dark** | 531 | 7965 | 132,75 | 16 | 11 |
| **Boss** | 476 | 7140 | 119 | 21 | 12 |
| **Cry** | 439 | 6585 | 109,75 | 13 | 9 |
| **Bruce** | 381 | 5715 | 95,25 | 22 | 11 |
| **Alone** | 357 | 5355 | 89,25 | 5 | 4 |
| **Ulet** | 287 | 4305 | 71,75 | 10 | 10 |
| **Mark** | 238 | 3570 | 59,5 | 19 | 11 |
| **Wave** | 231 | 3465 | 57,75 | 16? | 10 |
| **Dalì** | 231 | 3465 | 57,75 | 17 | 11 |
| **Amu1** | 146 | 2190 | 36,5 | 9 | 9 |
| **Kabbe** | 19 | 285 | 4,75 | ? | ? |

**Table S1.** Data on the different group units followed during the study, including the alfa male name, the number of scans in which the group was present, the observation time in minutes and hours, and the size of the group unit comprising all subjects, or only considering adult animals.

| **Behaviour** | **Description** |
| --- | --- |
| Avoidance | An individual retreats when it is displaced, threatened, or attacked. |
| Bite | An individual bites another, provoking reactions such as fleeing, crouching, screaming, or counterattacking. |
| Chase | An individual chases a fleeing counterpart, |
| Displacement | One individual moves toward or makes contact with another, prompting the latter to move away simultaneously. |
| Grab | An individual forcefully grabs or pulls another. |
| Open mouth bared-teeth scream | The mouth is opened wide with the corners drawn back, revealing the teeth and sometimes the gums. This facial expression is usually accompanied by direct staring and loud vocalizations (e.g., screams, geckers) and is commonly exhibited by victims of aggression. |
| Pushing | One individual pushes another using the hand or body, imparting momentum; the recipient may either resist or be displaced. |
| Run Away | Fleeing from another individual. |
| Eye-lid exposure (often associated with jaw fencing, bared-teeth, and lip-flip displays). | Exposure of the pale skin above the eyelids, often occurring alongside threat-related expressions such as the jaw-fence posture. This can accompany displays that don’t consistently signal aggression on their own, such as the bared-teeth expression (where the upper or both lips are vertically retracted to expose the teeth and sometimes gums, with mouth corners possibly pulled back and the jaw in varying positions) or the lip-flip display (Lazow & Bergman, 2020). |
| Slap | An individual strikes another using the flat of its hand. |
| Threat grunts | A short, staccato vocalization produced by a dominant individual during aggressive interactions (Gustison et al., 2012). |
| Attempt to bite | An animal attempts to bite a peer but fails to make contact. |
| Attempt to steal infant | An individual tries, whether successfully or not, to abduct an infant from a female—this behavior is usually exhibited by subadult or young adult females. |
| Wrestling | Two individuals engage in a physical fight. |

**Table S2.** Gelada agonistic behavioural patterns recorded during the observation period.

| **Behaviour** | **Description** |
| --- | --- |
| Grunt sequence | A soft, tonal contact call produced during approaches, grooming, infant-handling, as well as while moving or foraging. |
| Complex vocal sequence | A vocal sequence characterized by moaning and wobbling sounds. |
| Pre-copulation call | Vocalizations emitted by oestrous females while presenting their genitals to males. |
| Lip-smacking | Rapid lip movements, often accompanied by a clicking or smacking sound. |
| Bared-teeth lip-flip | A bared-teeth expression—where the upper or both lips are vertically retracted to reveal the teeth and sometimes gums, with the corners of the mouth drawn back and the jaw variably open or closed—frequently associated with the lip-flip movement, where the upper lip is lifted and everted (Lazow & Bergman, 2020). |
| Rear presenting | An individual raises or orients its hindquarters toward a nearby partner and may turn its head toward them. In more intense presentations (e.g., by estrous females), the individual lowers its head to the ground, flexes the arms, and elevates the rump. This may serve as an invitation for anogenital inspection, mounting, play, grooming, or may be a submissive response to threat. |
| Genital sniffing | Close visual, tactile, or olfactory examination of another individual's genital area, performed by a male or female. |
| Grooming | One individual grooms another by cleaning their skin or fur, brushing and parting the hair using the hands, and removing debris with the hands, mouth, teeth, or tongue. Grooming may be unidirectional or mutual. |
| Gentle touch | An individual makes gentle contact with another using the hand on a body area other than the hip. This affiliative gesture involves no pushing, grasping, or pulling. |
| Sit in contact | Two individuals sit in direct physical contact with one another. |
| Mount | One individual climbs onto the back of a standing partner from underneath (ventrodorsally). The mounter may or may not grasp the partner’s legs. This behaviour may indicate copulation or serve an affiliative purpose. |
| Social play | Playful interaction involving two or more individuals engaged in behaviours such as grabbing, pirouetting, pulling or pushing, slapping, and wrestling. |

**Table S3.** Behavioural affiliative patterns recorded during PC and MC observations.

**Supplementary Text**

**Coding Reliability.** We assessed intra-observer reliability on 15% (92 MC/PC observations randomly selected) of the videos which were re-coded by LP (minimum distance between the two coding: 35 days) using Cohen's Kappa coefficient. This was always higher than 0.85 (winner: *K* = 0.97; physical contact intensity of the agonistic event: *K* = 0.86; victim screams: *K* = 0.91; presence and type of third-party intervention: *K* = 0.85; presence and modality of conciliatory signal(s): *K* = 0.91; type of conciliatory pattern(s) used: *K* = 0.88; victim and aggressor scratching: *K_mean_* = 0.88). LP also re-coded the ID of subjects involved in the subset of aggression, with 97% of agreements between the two codlings.

**Dominance hierarchy.** We used the Average Dominance Index (Saccà et al., 2022) to derive intra-group-unit ranking scores of the recognized subjects of the OMUs. This index, suitable for linear hierarchies as those formed in gelada groups (le Roux et al., 2011), overcomes substantial biases in the steepness of hierarchies, especially when numerous dyads within the groups exhibit no interactions (typical when studying large groups) (Saccà et al., 2022). All aggressive events (overt aggression and threat/avoidance interactions) without clear winner/loser roles were excluded.

**Social bond.** We recorded the identity of the individuals involved in each grooming interaction and the exact start and end times of each bout. A social grooming bout was defined as an interaction with no pauses longer than 10 seconds (Palagi et al., 2014). We assessed the strength of social bonds for each dyad by calculating a Composite Sociality Index (CSI) based on grooming, following (Silk et al., 2006). For each dyad, the CSI was first computed as the total time the two individuals spent grooming each other (in seconds), divided by the total observation time of their OMU, derived from 15-minute scan sampling. To allow for cross-dyad and cross-team comparisons, we normalized the resulting value in two steps: first, by dividing it by the average CSI of all dyads within the same OMU; and second, by dividing it again by the average CSI across all OMUs belonging to the same site. This index reflects the extent to which a dyad grooming rate deviates from the local (OMU-level) and broader (team-level) social average, providing a standardized measure of bond strength.

**Genetic analyses and kin relationships.**

**DNA Extraction**

Fresh faecal samples were collected from 112 subjects and preserved in 15 ml DNA/RNA Shield™ Fecal Collection tubes (Zymo Research), which stabilize nucleic acids, inactivate pathogens, and allow safe transport for subsequent DNA extraction and genotyping. Genomic DNA (gDNA) was extracted in a column using a silica membrane, capable of binding and retaining DNA, in the presence of chaotropic salts in the lysis buffer. For this purpose, 100μl of fecal material were subjected to genomic DNA extraction using a specific kit (QIAamp Fast DNA Stool Mini Kit – Qiagen), in which a specific buffer efficiently removes PCR inhibitors commonly present in stool samples.

**Genotyping and Kinship analysis**

For individuals genotyping, a set of 17 short tandem repeats (STRs) was used to define the genetic animal profile, as described by Miller and colleagues (Miller, Snyder-Mackler, et al., 2021). The amplification was organized in three different multiplex PCR (Table S4), each one first optimized with different primer annealing temperatures. In order to reduce allelic dropout and minimize typing error, we completed at least three independent PCRs per microsatellite set per individual (median 4 PCRs per microsatellite set per individual, range 3—6). One *locus* (D18s851) was excluded from the initial panel because it was not possible to amplify in more of the 50% of the individuals and, therefore, a panel of 16 *loci* was used. To avoid biases in the marker comparison caused by missing data, individuals screened at fewer than 75% of *loci* were excluded from the data set, therefore all individuals were genotyped at least for 12 *loci*. While this reduced our sample size of 10 individuals, it was deemed necessary to ensure high-quality data for robust kinship inference. Of the initial 112 individuals, 102 individuals (47 site Godo and 55 for Sett Deber site) were submitted to analysis. To test the robustness of amplification and genotyping method, null alleles frequencies and genotyping errors were estimated. Microsatellite genotyping error was estimated through COLONY2 (Version 2.0.7.0 Wang, 2004) software by simulating replicates, separating allelic dropout errors and other types of error. Null allele frequencies were estimated using the *null.all* function from the PopGenReport R-package, with 1000 bootstrap replicates, using Brookfield (1996) method and evaluated according to the criteria outlined by Adamack & Gruber (2014). According to these criteria, if the values of the 97.5^th^ percentile include zero across all *loci*, it indicates that for each *locus* the frequency of null alleles does not significantly differ from zero. Specifically in this analysis, the 97.5^th^ percentile values included zero across all *loci* (range: –0.25 to 0.17); thus, all 16 *loci* were considered for analysis. Because there was not certain information about types of kinship among individuals, we first conducted a PCoA using *GeneAlEx 6.5* (Peakall & Smouse, 2006, 2012) to evaluate the distribution of individuals of the two sites based on their genetic distances. As there was a significant overlap of the distribution of individual alleles of the two sites along the two principal axis, allele frequencies of the two sites were calculated jointly.

First, we evaluate pairwise relatedness between individuals using the software ML-Relate (Kalinowski et al., 2006). This program reconstructs kinships first by calculating the Maximum Likelihood (ML) estimate of the genetic relatedness and then, for each pair of individuals, tests a series of discrete genealogical hypotheses (e.g., Parent-Offspring, Full-Siblings, Half-Siblings, Unrelated) against each other by calculating the likelihood ratio (LR) for two competing hypotheses. The outcome of the analysis consisted in pairwise relationships of the individuals -coefficients of relatedness and type of relationship- and were further tested for kinship and parentage using COLONY 2.0.7.1. (Jones & Wang, 2010) and CERVUS 3.0 (Kalinowski et al., 2007) software. The program COLONY2 implements a full-pedigree likelihood method that simultaneously infers sibship and parentage from multilocus genotype data, considering the likelihood of the entire population being jointly correct. This approach significantly improves the accuracy of relationship assignments as it relies in the joint likelihood instead of pairwise relation and handling polygamy mating system and inbreeding. Specifically, ten independent runs were performed. Because all individuals currently alive of the populations at the specific sites, irrespective of age or social group, were sampled and, as some old individuals have likely originated from mating of deceased ones and, considering not-sampled extra unit males, was assumed that 70% of parents were sampled (according to studies of (Miller, Snyder-Mackler, et al., 2021). For our study population, due to the human impact in the study area (Abie & Bekele, 2016; Pedruzzi et al., 2025b) we also assumed the possibility of inbreeding (Charpentier et al., 2007), as well as of a certain degree of polygamy for both sexes (Miller, Snyder-mackler, et al., 2021). Analyses were carried out without sex information for parents. In cases of discrepancies between the two methods, and assuming that COLONY2 is a more robust method for kinship assignment than ML-Relate, we further tested the specific hypothesis of relationship between the two individuals using maximum likelihood method with ML-Relate, simulating 10,000 genotypes. If the outcome of the specific hypothesis tests confirmed the outcome of the COLONY2 analysis, then the COLONY2 kinship relationship was assigned. In the rare cases in which the COLONY2 assignments weren’t confirmed and COLONY2 posterior probabilities of the assignments were low (cut-off values: <0.05), COLONY2 assignment was rejected. For parentage testing, in case of a Parent-Offspring (PO) assignment with *ML-Relate*, we used CERVUS3 software to confirm the relationship. CERVUS3 is a program that focuses specifically on assigning Parent-Offspring relationships (maternity, paternity and parent-pairs test). Primary it performs an exclusion analysis, based on Mendelian inheritance and then calculating Likelihood Ratio and LOD Score (LOD and Delta scores) for all the remaining, non-excluded candidate parents. The CERVUS3 simulations assumed 75% of parents sampled and 0.1% genotype error. If the LOD and Delta scores were positive, and, furthermore, a specific hypothesis tests through ML-Relate as described above, confirmed the Parent-Offspring relationship, PO was assigned.

The final outcome of these procedures classified each dyad as full siblings, half siblings, parent–offspring, or unrelated, and these categories were used in the subsequent analyses. Considering the dyads of subjects who have been opponents during conflicts, genetic profiling and relatedness of subjects from the different group units across sites generally confirmed some degree of relatedness among females and unrelatedness between males and females of the same group unit. However, exceptions were not negligible in some cases: in n=8 group units we found at least one dyad of unrelated females and/or the male being half-sibling with at least one female, and in n=2 group units (Small, Site 1; Medium, Site 2), we even found the male to be half-sibling with most of the adult females, who, on the other hand, were often unrelated with each other. Interestingly, two adult follower males living with the alpha male of two OMUs (i.e., always observed with the OMU) were unrelated with the alpha male but kin to several females of the group unit; moreover, subadult males living in the same group unit were always unrelated with each other and with the alpha male. These aspects can be due to limitation of the analysis as for some individuals complete genotype profile was not available as there was the presence of not-determined alleles or up to 3 complete not-amplified *loci*. It either raise interesting questions for further research on the benefit of allowing a unrelated male in the group unit and on the pattern of exogamy (Snyder-Mackler et al., 2012b), but also on violation of species sociobiology in human-impacted environments (Giuntini & Pedruzzi, 2023).

**Bayesian Modelling**

We conducted analyses within a Bayesian framework using the *brms* and *rstan* packages in R (Bürkner, 2017). Models were run through 4 Markov chains with 4000 (Model 1, 2a, 3a, 3b, 4a, 4b, 5a, 5b) or 8000 (Model 2b) iterations each (1000 warm-up for all models expect for Model 2b which had 2000 warm-up iterations), yielding 12,000 or 24,000 post-warmup draws. We set the *adapt_delta* control parameter to 0.95. To improve sampling efficiency and reduce the risk of divergent transitions for some models, we increased *adapt_delta* to or above 0.99 (Model 2b, 3a, 3b, 4a, 4b, 5a, 5b) and the maximum tree depth was increased to 15 to ensure full posterior exploration (Model 2a, 2b, 4a, 4b, 5b). We specified weakly informative priors. In most models, we used a normal(0, 1) prior for fixed effects and an exponential(1) prior for group-level standard deviations. In some cases (Model 2b, 3b, 4b, 5b), to allow for greater flexibility, we used wider priors: normal(0, 2) for fixed effects, normal(0, 5) for the intercept, and exponential(1) for group-level standard deviations (Grampp et al., 2023; Heesen et al., 2021; Mine et al., 2022). Model diagnostics based on Hamiltonian Monte Carlo (HMC) indicated proper convergence and efficient sampling: no divergent transitions occurred across the iterations, the maximum tree depth (10) was never exceeded, and energy diagnostics (E-BFMI) revealed no pathological behaviour. All parameters had Rhat values < 1.01, and effective sample sizes (ESS) were consistently high, supporting the reliability of posterior estimates. Model fit was further assessed through posterior predictive checks using the *pp_check()* function. Numeric variables were z-transformed with a mean of zero and a standard deviation of one, in order to more easily interpret their estimates. When the 95% of the credible interval of an estimate did not overlap 0, we considered that effect was supported by our data (Grampp et al., 2023). We reported p+ and p− as the percentages of posterior distribution in support for the direction of the estimate. We fitted Bayesian models and report parameter estimates as posterior means together with their 95% credible intervals (CI). **p+** indicates the posterior probability that a parameter is greater than zero (i.e., the proportion of the posterior distribution above zero). Values of p+ close to 1 indicate strong support for a positive effect, whereas values close to 0 indicate support for a negative effect. Because p+ reflects the proportion of the posterior distribution on one side of zero, it can occasionally be high even when the 95% credible interval slightly overlaps zero, particularly when the posterior distribution is skewed. Therefore, p+ should be interpreted as a measure of directional support rather than as a direct equivalent of frequentist p-values.

**References**

Abie, K., & Bekele, A. (2016). Threats to Gelada Baboon (Theropithecus gelada) around Debre Libanos, Northwest Shewa Zone, Ethiopia. *International Journal of Biodiversity*, *2016*, 1–7. https://doi.org/10.1155/2016/3405717

Adamack, A. T., & Gruber, B. (2014). PopGenReport: simplifying basic population genetic analyses in R. *Methods in Ecology and Evolution*, *5*(4), 384–387. https://doi.org/10.1111/2041-210X.12158

Brookfield, J. F. Y. (1996). A simple new method for estimating null allele frequency from heterozygote deficiency. *Molecular Ecology*, *5*(3), 453–455. https://doi.org/10.1046/j.1365-294X.1996.00098.x

Bürkner, P.-C. (2017). brms : An R Package for Bayesian Multilevel Models Using Stan. *Journal of Statistical Software*, *80*(1). https://doi.org/10.18637/jss.v080.i01

Charpentier, M. J. E., Widdig, A., & Alberts, S. C. (2007). Inbreeding depression in non‐human primates: a historical review of methods used and empirical data. *American Journal of Primatology*, *69*(12), 1370–1386. https://doi.org/10.1002/ajp.20445

Giuntini, S., & Pedruzzi, L. (2023). Sex and the patch: the influence of habitat fragmentation on terrestrial vertebrates’ mating strategies. *Ethology Ecology & Evolution*, *35*(3), 269–298. https://doi.org/10.1080/03949370.2022.2059787

Grampp, M., Samuni, L., Girard-Buttoz, C., León, J., Zuberbühler, K., Tkaczynski, P., Wittig, R. M., & Crockford, C. (2023). Social uncertainty promotes signal complexity during approaches in wild chimpanzees ( Pan troglodytes verus ) and mangabeys ( Cercocebus atys atys ). *Royal Society Open Science*, *10*(11). https://doi.org/10.1098/rsos.231073

Heesen, R., Bangerter, A., Zuberbühler, K., Iglesias, K., Neumann, C., Pajot, A., Perrenoud, L., Guéry, J. P., Rossano, F., & Genty, E. (2021). Assessing joint commitment as a process in great apes. *IScience*, *24*(8). https://doi.org/10.1016/j.isci.2021.102872

Jones, O. R., & Wang, J. (2010). COLONY: a program for parentage and sibship inference from multilocus genotype data. *Molecular Ecology Resources*, *10*(3), 551–555. https://doi.org/10.1111/j.1755-0998.2009.02787.x

Kalinowski, S. T., Taper, M. L., & Marshall, T. C. (2007). Revising how the computer program cervus accommodates genotyping error increases success in paternity assignment. *Molecular Ecology*, *16*(5), 1099–1106. https://doi.org/10.1111/j.1365-294X.2007.03089.x

Kalinowski, S., Wagner, A. P., & Taper, M. L. (2006). ml-relate: a computer program for maximum likelihood estimation of relatedness and relationship. *Molecular Ecology Notes*, *6*(2), 576–579. https://doi.org/10.1111/j.1471-8286.2006.01256.x

le Roux, A., Beehner, J. C., & Bergman, T. J. (2011). Female philopatry and dominance patterns in wild geladas. *American Journal of Primatology*, *73*(5), 422–430. https://doi.org/10.1002/ajp.20916

Miller, C. M., Snyder-mackler, N., Nguyen, N., Fashing, P. J., Tung, J., Wroblewski, E. E., Gustison, M. L., & Wilson, M. L. (2021). Extragroup paternity in gelada monkeys , Theropithecus gelada , at Guassa , Ethiopia and a comparison with other primates. *Animal Behaviour*, *177*, 277–301. https://doi.org/10.1016/j.anbehav.2021.05.008

Miller, C. M., Snyder-Mackler, N., Nguyen, N., Fashing, P. J., Tung, J., Wroblewski, E. E., Gustison, M. L., & Wilson, M. L. (2021). Extragroup paternity in gelada monkeys, Theropithecus gelada, at Guassa, Ethiopia and a comparison with other primates. *Animal Behaviour*, *177*, 277–301. https://doi.org/10.1016/j.anbehav.2021.05.008

Mine, J. G., Slocombe, K. E., Willems, E. P., Gilby, I. C., Yu, M., Thompson, M. E., Muller, M. N., Wrangham, R. W., Townsend, S. W., & Machanda, Z. P. (2022). Vocal signals facilitate cooperative hunting in wild chimpanzees. *Science Advances*, *8*(30). https://doi.org/10.1126/sciadv.abo5553

Palagi, E., Dall’Olio, S., Demuru, E., & Stanyon, R. (2014). Exploring the evolutionary foundations of empathy: consolation in monkeys. *Evolution and Human Behavior*, *35*(4), 341–349. https://doi.org/10.1016/j.evolhumbehav.2014.04.002

Peakall, R., & Smouse, P. E. (2006). genalex 6: genetic analysis in Excel. Population genetic software for teaching and research. *Molecular Ecology Notes*, *6*(1), 288–295. https://doi.org/10.1111/j.1471-8286.2005.01155.x

Peakall, R., & Smouse, P. E. (2012). GenAlEx 6.5: genetic analysis in Excel. Population genetic software for teaching and research—an update. *Bioinformatics*, *28*(19), 2537–2539. https://doi.org/10.1093/bioinformatics/bts460

Pedruzzi, L., Galotti, A., Francesconi, M., Quartesan, A., Gamessa, S. A., Bogale, B. A., Petroni, G., Serra, V., Lemasson, A., & Palagi, E. (2025). Behavioural adaptability of wild geladas (Theropithecus gelada) in human-impacted areas revealed by encounters with free-roaming dogs and potential threats. *Global Ecology and Conservation*, *62*, e03738. https://doi.org/10.1016/J.GECCO.2025.E03738

Saccà, T., Gort, G., van de Waal, E., & Hemelrijk, C. K. (2022). Reducing the bias due to unknown relationships in measuring the steepness of a dominance hierarchy. *Animal Behaviour*, *193*, 125–131. https://doi.org/10.1016/j.anbehav.2022.09.002

Silk, J. B., Alberts, S. C., & Altmann, J. (2006). Social relationships among adult female baboons (Papio cynocephalus) II. Variation in the quality and stability of social bonds. *Behavioral Ecology and Sociobiology*, *61*(2), 197–204. https://doi.org/10.1007/s00265-006-0250-9

Snyder-Mackler, N., Alberts, S. C., & Bergman, T. J. (2012). Concessions of an alpha male? Cooperative defence and shared reproduction in multi-male primate groups. *Proceedings of the Royal Society B: Biological Sciences*, *279*(1743), 3788–3795. https://doi.org/10.1098/rspb.2012.0842

Wang, J. (2004). Sibship Reconstruction From Genetic Data With Typing Errors. *Genetics*, *166*(4), 1963–1979. https://doi.org/10.1093/genetics/166.4.1963

| PCR mix | Primer | Forward (5’-3’) | Reverse (5’-3’) |
| --- | --- | --- | --- |
| 2.2  (58°C) | D8s1106 | FAM - TTGTTTACCCCTGCATCACT | TTCTCAGAATTGCTCATAGTGC |
|  | D4s243 | VIC - TCAGTCTCTCTTTCTCCTTGCA | TAGGAGCCTGTGGTCCTGTT |
|  | D6s501 | NED - CTGGAAACTGATAAGGGCT | GCCACCCTGGCTAAGTTACT |
|  | D6s291 | FAM - CTCAGAGGATGCCATGTCTAAAATA | GGGGATGACGAATTATTCACTAACT |
|  | D18s851 | VIC - CTGTCCTCTAGGCTCATTTAGC | TTATGAAGCAGTGATGCCAA |
|  | D11s2002 | NED - CATGGCCCTTCTTTTCATAG | AATGAGGTCTTACTTTGTTGCC |
| 3.3  (56°C) | D5s1457 | FAM - TAGGTTCTGGGCATGTCTGT | TGCTTGGCACACTTCAGG |
|  | AGAT006 | VIC - AGTGGATCGATAGATTGACAGATG | TCAGGTGACAGCCAAGTCAATTCA |
|  | D18s536 | NED - ATTATCACTGGTGTTAGTCCTCTG | CACAGTTGTGTGAGCCAGTC |
|  | D14s306 | PET - AAAGCTACATCCAAATTAGGTAGG | TGACAAAGAAACTAAAATGTCCC |
|  | D3s1766 | FAM - ACCACATGAGCCAATTCTGT | ACCCAATTATGGTGTTGTTACC |
|  | D6s1960 | VIS - GCCTCTCCTTCTTCACTTCC | CTCAACAACAACAAATGTAGCA |
| 4.1  (58°C) | D17s791 | FAM - GTTTTCTCCAGTTATTCCCC | GCTCGTCCTTTGGAAGAGTT |
|  | D5s111 | VIC - GGCATCATTTTAGAAGGAAAT | ACATTTGTTCAGGACCAAAG |
|  | D3s1768 | NED - GGTTGCTGCCAAAGATTAGA | CACTGTGATTTGCTGTTGGA |
|  | D1s548 | PET - GAACTCATTGGCAAAAGGAA | GCCTCTTTGTTGCAGTGATT |
|  | D6s311 | FAM - ATGTCCTCATTGGTGTTGTG | GATTCAGAGCCCAGGAAGAT |

**Table S4.** List of 17 short tandem repeats (STRs) used to define individual genetic profiles, as described in Miller et al. (2021). The STRs were amplified using three separate multiplex PCR reactions. The table includes the primer sequences grouped by PCR mix, the corresponding annealing temperatures, and the fluorescent label associated with each forward primer.

| **Signals in sequence** | **N° of cases in PC observations** |
| --- | --- |
| Complex vocal sequence | 77 |
| Grunts | 42 |
| Rear-presenting | 17 |
| Lip-smacking | 12 |
| Complex vocal sequence, lip-smacking | 7 |
| Grooming | 6 |
| Rear-presenting, grooming | 5 |
| Lip-smacking, grooming | 4 |
| Lip-smacking, complex vocal sequence | 4 |
| Lip-smacking, grunts | 3 |
| Complex vocal sequence, lip-smacking, gentle touch | 3 |
| Bared-teeth lip-flip | 3 |
| Pre-copulation call, rear-presenting | 3 |
| Complex vocal sequence, lip-smacking, mounting | 2 |
| Lip-smacking, complex vocal sequence, genital sniffing | 2 |
| Complex vocal sequence, gentle touch | 2 |
| Body contact | 2 |
| Social play, play face | 2 |
| Rear-presenting, pre-copulation call | 2 |
| Bared-teeth lip-flip, rear-presenting | 2 |
| Lip-smacking, rear-presenting | 1 |
| Lip-smacking, gentle touch | 1 |
| Complex vocal sequence, genital sniffing | 1 |
| Lip-smacking, complex vocal sequence, mounting | 1 |
| Play face, social play, grooming | 1 |
| Grunts, gentle touch | 1 |
| Bared-teeth lip-flip, complex vocal sequence | 1 |
| Lip-smacking, mounting | 1 |
| Gentle touch, grunts | 1 |
| Complex vocal sequence, lip-smacking, grooming | 1 |
| Rear-presenting, lip-smacking | 1 |
| Pre-copulation call | 1 |
| Play face, social play, rear-presenting | 1 |
| Lip-smacking, bared-teeth lip-flip | 1 |
| Bared-teeth lip-flip, pre-copulation call | 1 |
| Rear-presenting, grooming | 1 |

**Table S5.** Data on the variability of patterns used in post conflict observations (including sequences produced by the first signaller and, eventually, by the second subject).

| **Modalities** | **N° of cases in PC observations** |
| --- | --- |
| Tactile, acoustic | 5 |
| Tactile, visual, acoustic | 6 |
| Tactile | 8 |
| Tactile, visual | 19 |
| Visual, acoustic | 21 |
| Visual, acoustic | 37 |
| Acoustic | 120 |

**Table S6.** Data on the variability of modalities used in post conflict observations (including sequences produced by the first signaller and, eventually, by the second subject).

| **Model 1a. Predictors.** | **Estimate** | **95% CrI** | **Supported effect** |
| --- | --- | --- | --- |
| Intercept | 16.04 | [14.28, 17.88] | — |
| Victim age (juvenile) | -0.17 | [-1.57, 1.26] | No |
| Victim age (subadult) | -0.17 | [-1.39, 1.07] | No |
| Aggressor age (juvenile) | 0.55 | [-1.26, 2.38] | No |
| **Aggressor age (subadult)** | **1.84** | **[0.32, 3.37]** | **Yes** |
| Victim sex (male) | 1.05 | [-0.21, 2.30] | No |
| **Aggressor sex (male)** | **-4.36** | **[-5.43, -3.25]** | **Yes** |
| **Condition (PC)** | **-6.55** | **[-7.50, -5.63]** | **Yes** |
| OMU adults | 0.17 | [-0.56, 0.81] | No |
| **Adults in proximity** | **-0.50** | **[-0.91, -0.09]** | **Yes** |
| Anthropogenic impact (livestock) | -0.20 | [-1.30, 0.91] | No |
| Anthropogenic impact (humans + livestock) | -0.58 | [-1.70, 0.55] | No |
| n_observations_=610; n_days_=73; n_aggressors_=55; n_victim_=81; n_OMUs_=14; R² =0.5 (censored model) | | | |

**Table S7.** Bayesian Model 1a full results

| **Model 1b. Predictors** | **Estimate** | **95% CrI** | **Supported effect** |
| --- | --- | --- | --- |
| Intercept | 16.02 | [14.29, 17.80] | — |
| Victim age (juvenile) | -0.17 | [-1.59, 1.26] | No |
| Victim age (subadult) | -0.18 | [-1.40, 1.04] | No |
| Aggressor age (juvenile) | 0.56 | [-1.21, 2.35] | No |
| **Aggressor age (subadult)** | **1.84** | **[0.31, 3.35]** | **Yes** |
| Victim sex (male) | 1.07 | [-0.21, 2.30] | No |
| **Aggressor sex (male)** | **-4.36** | **[-5.43, -3.26]** | **Yes** |
| **Condition (PC)** | **-6.55** | **[-7.48, -5.64]** | **Yes** |
| OMU adults | 0.17 | [-0.54, 0.81] | No |
| **Adults in proximity** | **-0.50** | **[-0.91, -0.09]** | **Yes** |
| Anthropogenic impact (livestock) | -0.20 | [-1.30, 0.91] | No |
| Anthropogenic impact (humans + livestock) | -0.57 | [-1.69, 0.54] | No |
| n_observations_=610; n_days_=88; n_aggressors*victim_=197; n_OMUs_=15; R² =0.5 (censored model) | | | |

**Table S8.** Bayesian Model 1b full results

| **Model 1c. Predictors** | **Estimate** | **95% CrI** | **Supported effect** |
| --- | --- | --- | --- |
| Intercept | 16.03 | [14.30, 17.85] | — |
| Victim age (juvenile) | -0.16 | [-1.56, 1.27] | No |
| Victim age (subadult) | -0.16 | [-1.40, 1.08] | No |
| Aggressor age (juvenile) | 0.55 | [-1.27, 2.33] | No |
| Aggressor age (subadult) | 1.84 | [0.34, 3.36] | Yes |
| Victim sex (male) | 1.06 | [-0.20, 2.31] | No |
| Aggressor sex (male) | -4.37 | [-5.48, -3.26] | Yes |
| Condition (PC) | -6.55 | [-7.50, -5.64] | Yes |
| OMU adults | 0.16 | [-0.55, 0.81] | No |
| Adults in proximity | -0.51 | [-0.92, -0.11] | Yes |
| Anthropogenic impact (livestock) | -0.20 | [-1.32, 0.94] | No |
| Anthropogenic impact (humans + livestock) | -0.58 | [-1.70, 0.54] | No |
| n_observations_=610; n_days_=88; n_aggressors*victim_=197; n_OMUs_=15; R² =0.5 (censored model) | | | |

**Table S9.** Bayesian Model 1c full results

| **Model 1d. Predictors** | **Estimate** | **95% CrI** | **Supported effect** |
| --- | --- | --- | --- |
| Intercept | 16.04 | [14.28, 17.88] | — |
| Victim age (juvenile) | -0.17 | [-1.59, 1.28] | No |
| Victim age (subadult) | -0.16 | [-1.36, 1.03] | No |
| Aggressor age (juvenile) | 0.57 | [-1.21, 2.37] | No |
| **Aggressor age (subadult)** | **1.84** | **[0.33, 3.37]** | **Yes** |
| Victim sex (male) | 1.05 | [-0.20, 2.28] | No |
| **Aggressor sex (male)** | **-4.36** | **[-5.45, -3.24]** | **Yes** |
| **Condition (PC)** | **-6.56** | **[-7.48, -5.65]** | **Yes** |
| OMU adults | 0.18 | [-0.52, 0.82] | No |
| **Adults in proximity** | **-0.50** | **[-0.91, -0.10]** | **Yes** |
| Anthropogenic impact (livestock) | -0.21 | [-1.33, 0.93] | No |
| Anthropogenic impact (humans + livestock) | -0.59 | [-1.71, 0.56] | No |
| n_observations_=610; n_days_=88; n_aggressors*victim_=197; n_OMUs_=15; R² =0.5 (censored model) | | | |

**Table S10.** Bayesian Model 1d full results

| **Model 2a. Predictors.** | **Estimate** | **95% CrI** | **Supported effect** |
| --- | --- | --- | --- |
| Intercept | -2.76 | [-6.02, -0.72] | — |
| Agonistic support (levelling) | -2.04 | [-5.28, 0.24] | No |
| Agonistic support (polarizing) | -0.46 | [-4.10, 3.04] | No |
| Aggression duration | -1.05 | [-2.69, 0.06] | No |
| Victim scream duration | 0.59 | [-0.14, 1.54] | No |
| Physical contact | -0.33 | [-1.44, 0.66] | No |
| Social bond | 0.40 | [-0.28, 1.13] | No |
| Rank difference | -0.75 | [-2.19, 0.15] | No |
| **Aggressor sex (male)** | **6.11** | **[3.28, 11.76]** | **Yes** |
| Victim sex (male) | -1.74 | [-5.19, 0.84] | No |
| **Aggressor age (subadult)** | **-3.61** | **[-9.08, -0.14]** | **Yes** |
| Victim age (subadult) | 0.59 | [-1.72, 3.24] | No |
| n_observations_=255; n_days_=73; n_aggressors_=55; n_victim_=81; n_OMUs_=14; R² = 0.68, 95 % CI [0.58, 0.79] | | | |
| **Model 2b. Predictors.** | **Estimate** | **95% CrI** | **Supported effect** |
| Intercept | -2.42 | [-4.52, -0.50] | — |
| Agonistic support (levelling) | -0.92 | [-2.80, 0.89] | No |
| Agonistic support (polarizing) | 0.66 | [-1.84, 3.09] | No |
| Kin relationship (Unrelated) | 0.59 | [-0.96, 2.20] | No |
| **Aggression duration** | **-0.94** | **[-2.09, -0.02]** | **Moderate** |
| **Victim scream duration** | **0.79** | **[0.06, 1.62]** | **Yes** |
| Physical contact | -0.02 | [-1.09, 1.04] | No |
| Social bond | 0.16 | [-0.56, 0.99] | No |
| Rank difference | -0.40 | [-1.22, 0.47] | No |
| **Aggressor sex (male)** | **4.04** | **[2.14, 6.10]** | **Yes** |
| Victim sex (male) | -1.11 | [-3.03, 0.77] | No |
| Aggressor age (subadult) | -1.24 | [-3.81, 1.23] | No |
| Victim age (subadult) | 0.57 | [-1.29, 2.37] | No |
| n_observations_=174; n_days_=64; n_aggressors_=37; n_victims_=54; n_OMUs_=13; R² = 0.64, 95 % CI [0.55, 0.72] | | | |

**Table S11.** Bayesian Model 2a and 2b full results

| **Model 3a. Predictors.** | **Estimate** | **95% CrI** | **Supported effect** |
| --- | --- | --- | --- |
| Intercept | 1.87 | [1.31, 2.45] | — |
| Sex of the signaller (Male) | -0.16 | [-0.65, 0.35] | No |
| Role of the signaller (Victim) | -0.39 | [-0.93, 0.14] | No |
| Sex of the receiver (Male) | 0.34 | [-0.07, 0.75] | No |
| **Victim scream duration** | **0.14** | **[0.02, 0.25]** | **Yes** |
| Aggression duration | 0.05 | [-0.07, 0.17] | No |
| Physical contact | -0.01 | [-0.23, 0.21] | No |
| Social bond | 0.06 | [-0.11, 0.22] | No |
| Sex combination (same-sex, SS) | -0.38 | [-0.77, 0.02] | No |
| Rank difference | 0.16 | [-0.02, 0.33] | No |
| Social bond × Sex combination (SS) | -0.18 | [-0.44, 0.09] | No |
| Rank difference × Sex combination (SS) | 0.14 | [-0.14, 0.43] | No |
| n_observations_=126; n_days_=52; n_aggressors_=23; n_victims_=59; n_OMUs_=13; R² = 0.37, 95% CI [0.24, 0.50] | | | |
| **Model 3b. Predictors.** | **Estimate** | **95% CrI** | **Supported effect** |
| Intercept | 1.39 | [0.87, 1.92] | — |
| **Kin relationship (U)** | **0.49** | **[0.06, 0.89]** | **Yes** |
| Sex of the signaller (Male) | -0.12 | [-0.66, 0.40] | No |
| Sex of the receiver (Male) | -0.11 | [-0.52, 0.31] | No |
| **Victim scream duration** | **0.14** | **[0.00, 0.28]** | **Yes** |
| Social bond | 0.02 | [-0.16, 0.20] | No |
| **Rank difference** | **0.18** | **[0.01, 0.34]** | **Moderate** |
| n_observations_=82; n_aggressors_=17; n_victims_=39; R² = 0.3-, 95% CI [0.19, 0.55] | | | |

**Table S12.** Bayesian Model 3a and 3b full results

| **Model 4a. Predictors.** | | **Estimate** | **95% CrI** | **Supported effect** |
| --- | --- | --- | --- | --- |
| Intercept | | -6.44 | [-14.03, -1.09] | — |
| Sex of the signaller (Male) | | 3.53 | [-0.81, 9.51] | No |
| **Role of the signaller (Victim)** | | **6.84** | **[1.13, 16.47]** | **Yes** |
| Sex of the receiver (Male) | | -3.60 | [-9.40, 0.13] | No |
| **Victim scream duration** | | **-1.15** | **[-2.38, -0.16]** | **Yes** |
| Aggression duration | | -0.50 | [-1.81, 0.53] | No |
| Physical contact | | -0.41 | [-2.32, 1.28] | No |
| **Social bond** | | **1.20** | **[0.03, 2.56]** | **Yes** |
| Sex combination (same-sex, SS) | | 0.27 | [-4.04, 4.05] | No |
| **Rank difference** | | **1.42** | **[0.13, 2.99]** | **Yes** |
| Social bond × Sex combination (SS) | | 0.37 | [-1.97, 3.38] | No |
| Rank difference × Sex combination (SS) | | -1.22 | [-3.97, 1.24] | No |
| n_observations_=126; n_aggressors_=23; n_victims_=59; n_OMUs_=13; R² = 0.29, 95% CI [0.17, 0.47] | | | | |
| **Model 4b. Predictors.** | **Estimate** | **Lower CI** | **Upper CI** | **Supported effect** |
| Intercept | -2.82 | -6.12 | -0.17 | — |
| Kin relationship (Unrelated) | -0.04 | -1.96 | 1.91 | No |
| Sex of the signaller (Male) | 0.23 | -2.05 | 2.62 | No |
| Sex of the receiver (Male) | -0.19 | -2.32 | 1.85 | No |
| **Victim scream duration** | **-0.81** | **-1.81** | **0.07** | **Moderate** |
| Social bond | 0.32 | -0.74 | 1.38 | No |
| **Rank difference** | **1.02** | **0.00** | **2.23** | **Yes** |
| n_observations_=82; n_aggressors_=17; n_victims_=39; R² = 0.29, 95% CI [0.17, 0.47] | | | | |

**Table S13.** Bayesian Model 4a and 4b full results.

| **Model 5a. Predictors.** | | **Estimate** | **95% CrI** | **Supported effect** |
| --- | --- | --- | --- | --- |
| Intercept | | 2.74 | [2.06, 3.43] | — |
| Sex of the signaller (Male) | | 0.17 | [-0.44, 0.77] | No |
| Sex of the receiver (Male) | | 0.07 | [-0.44, 0.62] | No |
| Victim scream duration | | 0.10 | [-0.04, 0.24] | No |
| Role of the signaller (Victim) | | -0.16 | [-0.83, 0.49] | No |
| Aggression duration | | 0.02 | [-0.14, 0.17] | No |
| Physical contact | | -0.03 | [-0.30, 0.25] | No |
| **Social bond** | | **0.20** | **[0.00, 0.39]** | **Yes** |
| Sex combination (same-sex, SS) | | -0.22 | [-0.73, 0.24] | No |
| **Rank difference** | | **0.27** | **[0.05, 0.49]** | **Yes** |
| Social bond × Sex combination (SS) | | -0.28 | [-0.62, 0.07] | No |
| Rank difference × Sex combination (SS) | | 0.06 | [-0.30, 0.41] | No |
| n_observations_=126; n_aggressors_=23; n_victims_=59; n_OMUs_=13; R² = 0.29, 95% CI [0.17, 0.47] | | | | |
| **Model 5b. Predictors.** | **Estimate** | **Lower CI** | **Upper CI** | **Supported effect** |
| Intercept | 2.57 | 1.62 | 2.93 | — |
| **Kin relationship (Unrelated)** | **0.51** | **0.03** | **0.98** | **Yes** |
| Sex of the signaller (Male) | 0.22 | -0.40 | 0.82 | No |
| Sex of the receiver (Male) | -0.23 | -0.69 | 0.24 | No |
| Victim scream duration | 0.11 | -0.05 | 0.28 | No |
| Social bond | 0.02 | -0.19 | 0.24 | No |
| **Rank difference** | **0.28** | **0.08** | **0.47** | **Yes** |
| n_observations_=82; n_aggressors_=17; n_victims_=39; R² = 0.31, 95% CI [0.16, 0.46] | | | | |

**Table S14.** Bayesian Model 5a and 5b full results.
